# Rationalizing kinetic behaviors of isolated boron sites catalyzed oxidative dehydrogenation of propane

**DOI:** 10.1038/s41467-023-42403-2

**Published:** 2023-10-16

**Authors:** Hao Tian, Wenying Li, Linhai He, Yunzhu Zhong, Shutao Xu, Hai Xiao, Bingjun Xu

**Affiliations:** 1 College of Chemistry and Molecular Engineering, Peking University, Beijing, 100871 China; 2 Beijing National Laboratory for Molecular Sciences, Beijing, 100871 China; 3 Department of Chemistry and Key Laboratory of Organic Optoelectronics & Molecular Engineering of Ministry of Education, Tsinghua University, Beijing, 100084 China; 4 National Engineering Laboratory for Methanol to Olefins, Dalian National Laboratory for Clean Energy, iChEM (Collaborative Innovation Center of Chemistry for Energy Materials), Dalian Institute of Chemical Physics, Chinese Academy of Sciences, Dalian, 116023 China

**Keywords:** Heterogeneous catalysis, Chemical engineering

## Abstract

Boron-based catalysts exhibit high alkene selectivity in oxidative dehydrogenation of propane (ODHP) but the mechanistic understanding remains incomplete. In this work, we show that the hydroxylation of framework boron species via steaming not only enhances the ODHP rate on both B-MFI and B-BEA, but also impacts the kinetics of the reaction. The altered activity, propane reaction order and the activation energy could be attributed to the hydrolysis of framework [B(OSi≡)_3_] unit to [B(OSi≡)_3−*x*_(OH···O(H)Si≡)_*x*_] (*x* = 1, 2, “···” represents hydrogen bonding). DFT calculations confirm that hydroxylated framework boron sites could stabilize radical species, e.g., hydroperoxyl radical, further facilitating the gas-phase radical mechanism. Variations in the contributions from gas-phase radical mechanisms in ODHP lead to the linear correlation between activation enthalpy and entropy on borosilicate zeolites. Insights gained in this work offer a general mechanistic framework to rationalize the kinetic behavior of the ODHP on boron-based catalysts.

## Abstract (web summary)

The kinetic parameters in heterogeneous catalysis are informative in a mechanistic study. Here, the authors show the correlation between the coordination environment of isolated boron sites and the kinetics of oxidative dehydrogenation of propane.

## Introduction

Propylene (C_3_H_6_) is a fundamental building block of the chemical industry to produce bulk chemicals such as polypropylene, acrylonitrile, propylene oxide and acrylic acid^1^. Non-oxidative propane (C_3_H_8_) dehydrogenation (PDH) has been commercialized to meet the growing demand of C_3_H_6_, but the efficiency of PDH is limited by the unfavorable thermodynamics and coking-induced rapid catalyst deactivation^2^. Oxidative dehydrogenation of propane (ODHP), which introduces molecular oxygen (O_2_) to facilitate the C–H activation of propane, has the potential to overcome the challenges faced by PDH^3,4^. However, conventional ODHP catalysts, such as vanadium-based catalysts, exhibit poor alkene selectivity, which is caused by the deep oxidation of C_3_H_6_ to form carbon monoxide (CO) and carbon dioxide (CO_2_)^5^. The development of catalysts with high C_3_H_6_ yield and long-term stability is critical to make ODHP commercially viable.

ODHP activity of supported boron oxide (B_2_O_3_) catalysts has been recognized since the late 1980s, and the first application of hexagonal boron nitride (h-BN) after oxyfunctionalization was reported in 2016^6–8^. Distinct from vanadium-based catalysts, boron-based catalysts are able to suppress further oxidation of alkenes in the presence of O_2_^8,9^. A second-order dependence on the partial pressure of C_3_H_8_ (*p*_C3H8_) and the apparent activation energy (*E*_app_) higher than 150 kJ·mol^−1^ were observed on most boron-based catalysts (Table S1). The supra-linear reaction order of C_3_H_8_ cannot be rationalized by classical surface-mediated mechanisms in heterogeneous catalysis, which led to much research effort in understanding the catalytic mechanism. Hermans and co-workers found that the conversion rate of C_3_H_8_ was proportional to the volume of catalyst bed, rather than the catalyst loading, in the h-BN catalyzed ODHP, implying that a surface-mediated pathway is unlikely to be able to account for the majority of observed activity^10^. They proposed a combined surface and gas-phase radical reaction mechanism capable of rationalizing the product distribution of ODHP observed on h-BN^11^. Our recent kinetic study on oxidative co-dehydrogenation of ethane (C_2_H_6_) and C_3_H_8_ over h-BN found that the apparent second-order dependence on the partial pressure of alkane in ODHP could be rationalized by two roles played by the alkane, i.e., radical generator and reactant^12^. The high propylene selectivity of bulk boron-based catalysts in ODHP is likely due to the involvement of gas-phase radicals in activating propane, though the exact mechanism for suppressing the further conversion of propylene remains unclear^13–15^. The study using the magic angle spinning (MAS) ^11^B solid-state nuclear magnetic resonance (NMR) spectroscopy revealed that oxidized boron species, denoted as B(OH)_*x*_O_3−*x*_ (where *x* = 0–3), were gradually formed on h-BN in ODHP^16,17^. Similar oxidized boron species could also be formed on other boron-containing materials under ODHP conditions, and are likely responsible for the catalytic activity^18^.

The identities of active structure of isolated boron sites and reaction mechanism are less clear. Hermans and coworkers synthesized boron-substituted MWW (B-MWW) zeolites with negligible ODHP activity^19^. ^11^B NMR results showed that most framework boron atoms were in the trigonal coordination environment, which led to the conclusion of isolated boron sites being inactive. Meanwhile, B-MWW prepared by Lu and coworkers showed quite similar overall and deconvoluted NMR features and exhibited high ODHP activity^20^. Xiao and coworkers synthesized boron-substituted MFI- and BEA-type (B-MFI and B-BEA) zeolites with most framework boron atoms located in the tetrahedral coordination environment both in as-synthesized and spent samples^21^. These B-MFI and B-BEA catalysts showed remarkable ODHP activities and durability. A first-order kinetics with respect to *p*_C3H8_ was determined on both borosilicate zeolites. Combined with computational results, a surface-mediated mechanism was proposed on trigonally coordinated framework boron atoms in ODHP. Qiu et al. assigned aggregated boron species in incompletely crystallized B-MFI as the most active structure^22,23^. These inconsistent literature claims highlight the need for further mechanistic studies.

Determination of kinetic parameters, i.e., the reaction order of C_3_H_8_ and the apparent activation energy (*E*_app_), proves to be informative in mechanistic studies of boron-catalyzed ODHP. The likely involvement of gas-phase radicals, as well as the high reaction temperature, in ODHP makes the detection of surface intermediates challenging and less relevant. In contrast, C_3_H_8_ reaction order could serve as a diagnostic variable to identify the dominant type of mechanism at play. A first-order kinetics for C_3_H_8_ indicates a surface-mediated mechanism^24–26^, which is typical on vanadium-based catalysts. Most bulk boron-based catalysts exhibit a second-order reaction for C_3_H_8_, indicating the involvement of a gas-phase radical mechanism^11,12^. Our recent work showed that spatial confinement of B_2_O_3_ nanoparticles in mesoporous support could lead to C_3_H_8_ reaction orders up to 3^27^, which was attributed to the introduction of a branching radical pathway.

In this study, we investigated the correlation between the boron coordination environment and the ODHP kinetics. Fresh B-MFI with majority of boron species incorporated in the framework exhibits a first-order dependence on *p*_C3H8_ and *E*_app_ ranging from 100 to 120 kJ·mol^−1^. Steaming treatment gradually hydrolyzes the B–O–Si bonds in B-MFI, which proceeds from [B(OSi≡)_3_] to hydroxyl group substituted [B(OSi≡)_3−*x*_(OH···O(H)Si≡)_*x*_] (*x* = 1, 2), and enhances the ODHP activity by up to a factor of 9 on B-MFI. Computational results show that the hydroxylation of the isolated boron sites could stabilize potential gas-phase radical species derived from O_2_, facilitating the initiation of the radical chain mechanism. Similar observations were made on B-BEA. Degree of hydroxylation on framework boron species is proposed to be the key in understanding the kinetic behaviors of boron-containing zeolites after different pretrement conditions and in different ranges of *p*_C3H8_.

## Results

### Boron coordination and ODHP performance of the fresh B-MFI Zeolite

B-MFI was synthesized via a solvent-free crystallization method reported by Zhou et al.^21^ as conventional hydrothermal method was prone to forming non-framework boron species^28,29^. The as-prepared sample is referred to as fresh B-MFI below. Fresh B-MFI consists of sphere-like particles with an average diameter of ~1 μm (Fig. S1). XRD patterns of fresh B-MFI, H-ZSM-5 and Silicalite-1 exhibit diffraction peaks characteristic of the MFI-type zeolites with no detectable phase impurities. (Fig. S2) Lattice parameters and unit cell volumes of the MFI-type zeolites were calculated based on the peak positions in the XRD patterns. (Table S2) Compared to H-ZSM-5 and Silicalite-1, the unit cell volume of B-MFI is slightly contracted, indicating that the boron atoms are successfully incorporated into the framework of zeolite^30^. Micropore volume of fresh B-MFI, H-ZSM-5 and Silicalite-1 are in line with typical MFI-type zeolites^31^. (Fig. S3 and Table S3)

To understand the coordination environment of the boron species, ^11^B and ^29^Si NMR spectra of fresh B-MFI were collected. (Figs. S4 and S5) ^11^B chemical shift of fresh B-MFI shows a strong peak at −3.2 ppm and a broad peak at 5.9 ppm. The former has been assigned to the tetra-coordinated boron species in zeolite frameworks (denoted as B[4]-Fr)^32^. The band at −3.2 ppm exhibits a Lorentzian lineshape, which is in line with B[4]-Fr^33^. Distinct from tetrahedrally coordinated boron species, the strong quadrupolar interaction of trigonally coordinated boron leads to a peak shift and anisotropic line broadening of tri-coordinated boron species^34^. Combing the chemical shift and the lineshape, the peak at 5.9 ppm could be attributed to tri-coordinated boron species in the zeolite framework (denoted as B[3]-Fr)^35^. Non-framework tri-coordinated boron species (denoted as B[3]-Nf), for example H_3_BO_3_ (*δ*_iso_ = 18.0–18.5 ppm)^36,37^ and B_2_O_3_ (*δ*_iso_ = 14.6 ppm)^38^, are absent in fresh B-MFI, indicating that most boron atoms are incorporated in the framework and isolated by neighboring –O–Si–O– units. ^29^Si NMR spectrum of fresh B-MFI exhibits a strong peak at −113 ppm and a shoulder peak at −103 ppm, corresponding to [Si(OSi≡)_4_] and [Si(OSi≡)_3_OH] in the zeolite framework, respectively^39^. (Fig. S5) According to the peak area in Fig. S4, B[4]-Fr is estimated to account for 92% of all boron atoms at room temperature in fresh B-MFI, which is consistent with a previous study^21^.

**Fig. 1:**
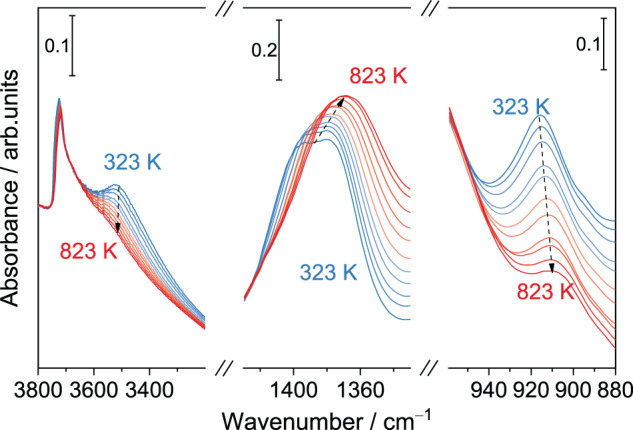
Influence of temperature on boron coordination. Transmission IR bands of OH groups (left part), trigonal framework boron species (middle part) and tetrahedral framework boron species (right part) on fresh B-MFI at different temperatures.

Although most framework boron atoms are in the form of B[4]-Fr at room temperature, in-situ IR spectroscopy shows that B[3]-Fr is the dominant species at reaction temperature. To study the boron coordination under the ODHP conditions, transmission IR spectra of dehydrated fresh B-MFI at different temperatures were collected. (Fig. S6) The peak around 3720 cm^−1^ is attributed to silanol groups (Si–OH) on zeolite and the peak at 3520 cm^−1^ is assigned to the Brønsted acid site in borosilicate zeolite^39,40^. (Fig. 1) The band at 910 cm^−1^ is attributed to the symmetric stretching of B[4]-Fr while the band located in the 1400 to 1360 cm^−1^ range corresponds to the B–O asymmetric stretching vibration of B[3]-Fr^34,41^. (Fig. 1) As temperature rises, the intensity of IR peaks at 3520 and 910 cm^−1^ decrease concomitantly while the peak at 1380 cm^−1^ strengthens, indicating that B[4]-Fr is transformed to B[3]-Fr at temperatures relevant in ODHP. Together with the absence of IR band for B–OH at 3680 cm^−1^ ^37,42^, it could be inferred that [B(OSi≡)_3_] unit is the dominant boron specie of fresh B-MFI at the ODHP temperature^21,29,41^.

**Fig. 2:**
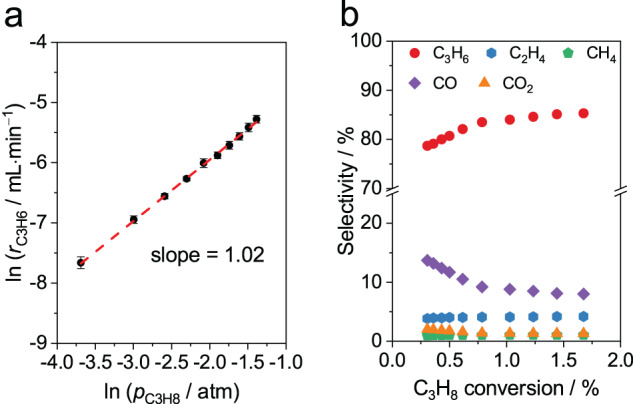
ODHP performance on fresh B-MFI. **a** The reaction order of *r*_C3H6_ with respect to *p*_C3H8_. Reaction conditions: 773 K, *p*_O2_ = 0.125 atm with balancing N_2_. Error bars show represent standard deviation of ln (*r*_C3H6_) in three replicate data. **b** Product selectivity as a function of C_3_H_8_ conversion. Reaction conditions: Reaction conditions: 773 K, *p*_C3H8_ = 0.25 atm, *p*_O2_ = 0.125 atm with balancing N_2_, space velocity from 1500 to 24000 L_C3H8_‧kg_cat_^−1^‧h^−1^.

Fresh B-MFI exhibits distinct catalytic behavior from most bulk boron-based catalysts in ODHP (Fig. 2a, b). To verify the initial ODHP activity prior to activation is attributed to framework boron species, we conducted two control experiments (Table S4 and Fig. S7). Silicalite-1 showed no ODHP selectivity to C_3_H_6_ (Table S4), and C_3_H_6_ formation rate of fresh B-MFI in ODHP exhibited a positive correlation to boron content in B-MFI (Fig. S7). The control experiments confirmed that the framework boron species are likely to be the active sites responsible for the initial ODHP activity in fresh B-MFI. The formation rate of C_3_H_6_ (*r*_C3H6_) over fresh B-MFI exhibits a first-order dependence on *p*_C3H8_. (Fig. 2a) This result is different from the supra-linear order observed over most bulk boron-based catalysts, but in line with a recent report on borosilicates^21^. (Table S1) Product distribution in ODHP on fresh B-MFI as a function of the C_3_H_8_ conversion is shown in Fig. 2b. The propylene selectivity increases almost linearly with the propane conversion when the conversion is below 1%, and then gradually levels off at conversion above 1.5% at 85%. While ethylene (C_2_H_4_) is the main by-product in ODHP on most bulk boron-based catalysts^43^, CO is the main by-product on fresh B-MFI, and its selectivity increases with decreasing C_3_H_8_ conversion. The high CO selectivity in ODHP on boron-substituted zeolites was also observed by Lin and co-workers^44^. The distinct C_3_H_8_ reaction order and product distribution on fresh B-MFI indicate that ODHP on fresh B-MFI likely proceeds via a distinct pathway from that on catalysts with aggregated boron species, e.g., B_2_O_3_ and h-BN.

### The promotion of ODHP activity via steaming treatment

**Fig. 3:**
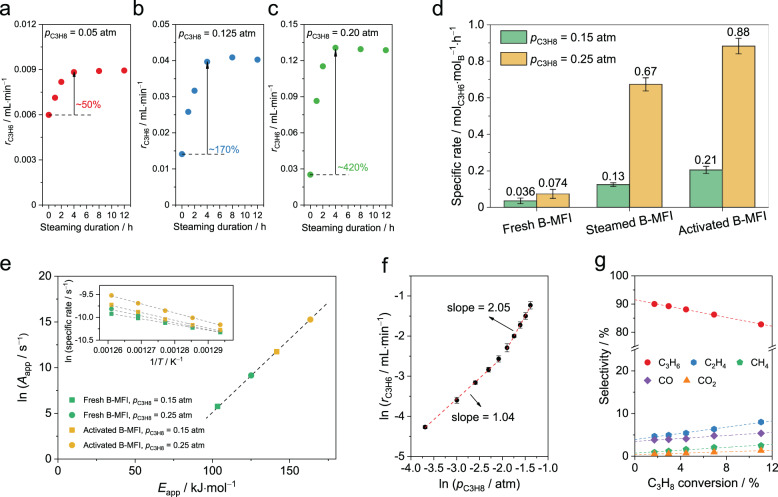
Influence of steaming on ODHP on B-MFI. **a**–**c** Influence of steaming duration on *r*_C3H6_ at different *p*_C3H8_: **a** *p*_C3H8_ = 0.05 atm; **b** *p*_C3H8_ = 0.125 atm; **c** *p*_C3H8_ = 0.20 atm. **d** Specific C_3_H_6_ formation rate of different B-MFI. Error bars show represent standard deviation of specific rates in three replicate data. Reaction conditions for (**a**–**d**): 803 K, total gas flow = 40 mL·min^−1^, 50 mg catalyst, *p*_O2_ = 0.125 atm with balancing N_2_. **e** Correlation between *E*_app_ and *A*_app_. The inset are corresponding Arrhenius plots. Reaction conditions for (**e**): 773 to 793 K, total gas flow = 40 mL·min^−1^, 50 mg catalyst, *p*_O2_ = 0.125 atm with balancing N_2_. **f** Reaction order of *r*_C3H6_ with respect to *p*_C3H8_ on activated B-MFI. Error bars show represent standard deviation of ln (*r*_C3H6_) in three replicate data. **g** Product selectivity (% of carbon atoms in C_3_H_8_ converted to products) as a function of C_3_H_8_ conversion on activated B-MFI. Reaction conditions for (**f**, **g**): 773 K, total gas flow = 40 mL·min^−1^, *p*_O2_ = 0.125 atm with balancing N_2_.

Steaming treatment markedly promotes the ODHP activity on fresh B-MFI. Steaming is an effective strategy to accelerate the hydrolysis of Si–O–X bonds (X represents a heteroatom) in zeolites, and thus tuning the coordination of framework heteroatoms such as boron^45,46^. The steaming of fresh B-MFI was carried out at high temperature (823 K) and low partial pressure of water (*p*_H2O_ = 1.8 kPa). *r*_C3H6_ increases almost linearly with the duration of the steaming treatment within the first 3 h and levels off after 4 h regardless of *p*_C3H8_ (Fig. 3a–c). The increase in the ODHP activity after steaming is more pronounced at high *p*_C3H8_, and the apparent reaction order of C_3_H_8_ increases with the steaming duration. (Fig. S8) The specific C_3_H_6_ formation rate on fully steamed B-MFI (with steaming duration > 4 h) reaches 0.67 mol_C3H6_·mol_B_^−1^·h^−1^ at *p*_C3H8_ = 0.25 atm and 803 K, which is nearly one order of magnitude higher than that of fresh B-MFI. (Fig. 3d) The ODHP activity of fully steamed B-MFI could be further enhanced in ODHP, i.e., there is an induction period^47^. (Fig. S9) Steamed B-MFI after the induction period is referred to as activated B-MFI later in this work. The fact that activated B-MFI exhibits superior ODHP activity than fully steamed sample indicates that species involved in the reaction are able to induce structrual changes at boron site that are inaccessible via steaming. The steady-state C_3_H_6_ formation rates after the induction period on fresh and fully steamed B-MFI are comparable, implying that ODHP could activate fresh B-MFI without the steaming treatment. This is expected considering that ODHP produces water, so the catalyst is subject to conditions similar to the steaming treatment during reaction. The specific C_3_H_6_ formation rate on activated B-MFI sample is 0.88 mol_C3H6_·mol_B_^−1^·h^−1^ at *p*_C3H8_ = 0.25 atm, (Fig. 3d) which is comparable to the PDH activity on noble metals^48^. To avoid interference of the induction period in determining the kinetic parameters of ODHP, activated B-MFI was employed in the following investigations.

Activated B-MFI exhibits a higher *E*_app_ than that on fresh B-MFI. (Fig. 3e and Table S5) At *p*_C3H8_ = 0.25 atm, *E*_app_ of activated and fresh B-MFI were determined to be 163 and 125 kJ·mol^−1^, respectively. These measured *E*_app_ values appear counterintuitive as the more active activated B-MFI exhibits a higher *E*_app_. The apparent pre-exponential factor (*A*_app_) of ODHP on activated B-MFI is higher than that of fresh B-MFI by a factor of ~400. Values of *E*_app_ and *A*_app_ also depend on *p*_C3H8_. When *p*_C3H8_ is decreased from 0.25 to 0.15 atm, both *E*_app_ and *A*_app_ of fresh and activated B-MFI are reduced, leading to a linear correlation between ln(*A*_app_) and *E*_app_ (Fig. 3e and Table S5). Literature suggest that *E*_app_ is typically higher with mechanisms involving gas-phase radicals (>150 kJ·mol^−1^) than that with surface-mediated mechanisms (< 120 kJ·mol^−1^)^27^. Thus, measured *E*_app_ values suggest that ODHP on activated B-MFI likely occurs via a radical mechanism.

C_3_H_8_ reaction order and product distribution in ODHP on activated B-MFI differ fundamentally from those on fresh B-MFI. The apparent C_3_H_8_ order on activated B-MFI increases from 1.04 to 2.05 as *p*_C3H8_ rises beyond 0.15 atm (Fig. 3f), while no such transition is present on fresh B-MFI (Fig. 2a). A first-order kinetics for C_3_H_8_ indicates that the reaction proceeds via a surface-mediated mechanism, while a second-order suggests a gas-phase radical mechanism^11,12,24–27^. This claim is also supported by the experiments that using silicon carbide (SiC) as a diluent to differentiate surface-mediated reactions from gas-phase radical reactions^10^. The introduction of SiC does not influence ODHP activity of fresh B-MFI, but markedly increases C_3_H_6_ formation rate of activated B-MFI (Fig. S10a). Enhancements in catalytic activity induced by diluent is a characteristic of gas-phase reaction, which could be attributed to the extended residence time of gas-phase active species (Fig. S10b). Moreover, similar to bulk boron species but distinct from fresh B-MFI, C_2_H_4_, rather than CO, is the main by-product on activated B-MFI. Further, the trends of C_3_H_6_ and CO selectivity with decreasing C_3_H_8_ conversion are opposite on fresh and activated B-MFI (Figs. 2b, 3g), indictive of distinct reaction mechanisms. C_3_H_8_ reaction order (*p*_C3H8_ > 0.15 atm) and product distribution on activated B-MFI are similar to those on catalysts with aggregated boron species, suggesting that the nature of active boron species on fresh B-MFI has been altered during the steaming treatment and the induction period.

### Evolution of active boron species in B-MFI

Characterization results show that elemental composition, morphology and crystalline structure of B-MFI remain largely unchanged during the steaming treatment and in the induction period. The melting point of B_2_O_3_ (723 K) is close to ODHP temperature and the volatilization of boron species was considered as a potential cause for catalyst deactivation^43^. B/Si molar ratios of both fresh and activated B-MFI determined by ICP-AES are consistent with that employed in the synthesis, indicating negligible boron loss. (Fig. S11) SEM images, XRD pattern and ^29^Si NMR spectra show that activated B-MFI retains the crystalline size, morphology, crystallographic parameters and silicon coordination environment after the steaming treatment and the induction period. (Figs. S12–14 and Table S6) Characterization results indicate that the enhanced ODHP activity on activated B-MFI is unlikely caused by changes in the compositional and structural change in the framework of B-MFI.

**Fig. 4:**
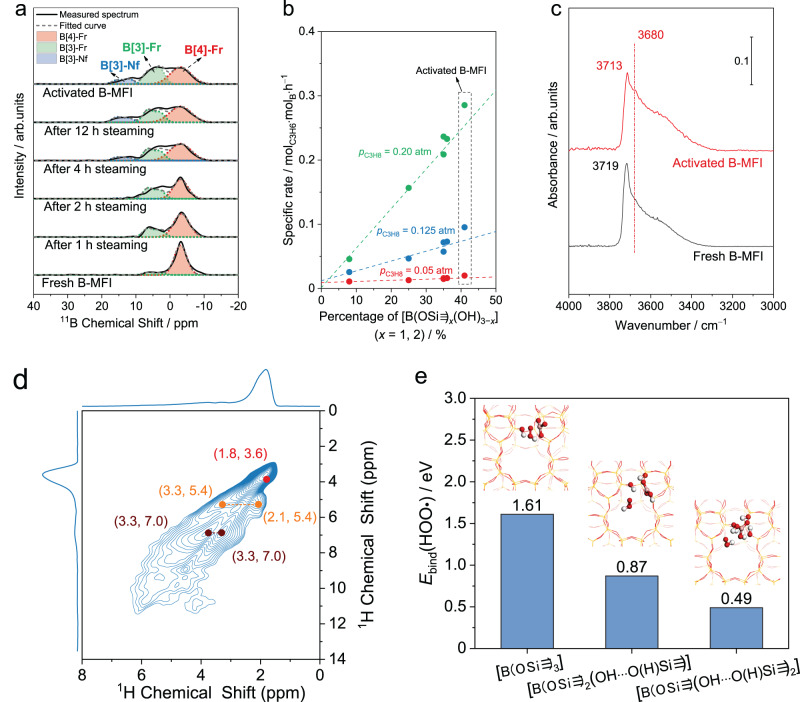
Transformation of boron species in B-MFI during steaming treatment and its impact on ODHP mechanism. **a** ^11^B NMR spectra and peak fitting of boron species on fresh, steamed and activated B-MFI. **b** Correlation between the specific ODHP activity and the percentage of [B(OSi ≡)_3−*x*_(OH)_*x*_] in B-MFI at various *p*_C3H8_ values. **c** Transmission IR spectra of dehydrated fresh and activated B-MFI under vacuum at 823 K. **d** 2D ^1^H-^1^H DQ MAS NMR spectrum of activated B-MFI. **e** Calcualted binding energy of HOO• on different boron sites. Balls and sticks in the inset pictures represent atoms: hydrogen (white), boron (pink), oxygen (red), and silicon (yellow).

^11^B NMR results indicate that the boron coordination in fresh B-MFI is gradually hydroxylated during the steaming treatment, but most boron atoms remain in the zeolite framework in fully steamed and activated B-MFI. ^11^B NMR spectra of various B-MFI samples were fitted with three peaks attributed to B[4]-Fr (red), B[3]-Fr (green) and B[3]-Nf (blue) (Fig. 4a, S15 and Table S7)^29,34,49^. B[4]-Fr is the dominant boron species (88%) in fresh B-MFI, with the rest being B[3]-Fr. With increasing steaming duration (1–4 h) at 823 K, an increasing fraction of B[4]-Fr is hydrolyzed to B[3]-Fr. B[3]-Fr bonded with hydroxyl, i.e., [B(OSi)_*x*_(OH)_3−*x*_] (*x* = 1, 2), are stabilized by the formation of hydrogen bonding network, suppressing the conversion of B[3]-Fr with hydroxyl to B[4]-Fr via condensation^34^. After steaming for 2 h, B[3]-Nf, i.e., B_2_O_3_ and hydroxylated B_2_O_*x*_(OH)_3−*x*_, appears in ^11^B NMR spectra due to the further hydrolysis of B–O–Si bond. The conversion from B[4]-Fr to B[3]-Fr, as well as the further hydroxylation of B[3]-Fr to B[3]-Nf, slows after 4 h of steaming. ODHP activity parallels the increase in the fraction of boron in B[3]-Fr within the first 4 h of steaming, and levels off afterwards (Fig. 3a–c). The fraction of boron species in the form of B[3]-Nf increases from 11% to 15% when B-MFI is steamed for additional 8 h after the initial 4 h, but with no appreciable change in the ODHP activity. The main difference in the distribution of various boron species between fully steamed and activated B-MFI is that B[3]-Fr accounts for a slightly higher fraction in activated B-MFI at the expense of B[4]-Fr (Fig. S15 and Table S7). The combined fraction of B[4]-Fr and B[3]-Fr in fully steamed and activated B-MFI is ≥ 85%, (Fig. S15 and Table S7) indicating that the majority of boron species are present as isolated in the zeolite framework. As discussed in Fig. 1, B[4]-Fr in ^11^B NMR spectra is converted into [B(OSi≡)_3_] at high temperature. It can be inferred that B[3]-Fr species, i.e., [B(OSi≡)_3−*x*_(OH)_*x*_] characterized by the ^11^B NMR spectroscopy, are largely responsible for the enhanced ODHP activity during steaming treatment. (Fig. 4b) The values of fitting lines on y-axis corresponds to the intrinsic activity of [B(OSi≡)_3_], which is the dominant boron specie on fresh B-MFI. As the duration of the steaming treatment increases, [B(OSi≡)_3_] is gradually hydroxylated to [B(OSi≡)_3−*x*_(OH)_*x*_], leading to a linear increase in the ODHP activity. (Fig. 4b) The slope of fitted lines in Fig. 4b, which is the growth rate of the ODHP activity with the content of [B(OSi≡)_3−*x*_(OH)_*x*_], is highly dependent on *p*_C3H8_. This is beacuse the supra-linear kinetics on steamed and activated B-MFI substantially enhances the ODHP rate at higher *p*_C3H8_. Due to the conversion of B[3]-Fr to B[4]-Fr induced by water uptake at room temperature^21,34^, the B[4]-Fr /B[3]-Fr ratios determined based on ^11^B NMR spectra collected at room temperature are likely overestimated compared to those at the reaction temperature, however, the increasing trend of the B[3]-Fr/B[4]-Fr ratio with the duration of the steaming treatment should hold regardless. We note that the observation that the steaming treatment itself is insufficient to fully activate B-MFI has been made on other boron-based catalysts in ODPH^50^. For example, the steaming treatment can only reduce the duration of, rather than replace, the induction period in ODHP (Fig. S9). Since the key difference between the steaming treatment and ODHP is the presence of reactive intermediates in the latter, we speculate that radical species generated in ODHP could help activate boron-based catalysts, though the exact mechanism remains unclear.

Hydroxylation of isolated boron sites during steaming is further supported by IR results. (Fig. 4c, S16 and S17) The intensity of the band attributable to intraporous B−OH groups with hydrogen bond interaction (~3680 cm^−1^)^21,51,52^ on activated B-MFI is stronger than that of fresh B-MFI at reaction temperature (823 K, Fig. 4c), which is strong evidence supporting the formation of B−OH group during the steaming treatment and the induction period. The peak located at 3710-3720 cm^−1^ is typically attributed to the silanol group on the intraporous and external surface of zeolite crystals^21,51,52^. Transmission IR spectra collected at different temperatures confirm that B[3]-Fr instead of B[4]-Fr is also the dominant boron species on activated B-MFI at elevated temperature, which is consistent with the discussion of fresh B-MFI. (Figs. S16 and S17) Combined with ^11^B NMR results, the appearance of B–OH group indicates that the isolated boron sites are derived from the hydroxylation of [B(OSi≡)_3_] to [B(OSi≡)_3−*x*_(OH)_*x*_]. (Fig. 4c and S18)

Results of ^1^H NMR experiments on activated B-MFI indicate the spatial proximity between boron hydroxyl in [B(OSi≡)_3−*x*_(OH)_*x*_] and silanol group. In the ^1^H MAS NMR spectrum of activated B-MFI (Fig. S19), ^1^H signals at 1.8, 2.1 and 3.1 ppm were observed, which could be assigned to terminal silanol groups (≡SiOH), silanol groups adjacent to oxygen (≡SiOH···O(H)X, X = Si≡ or B= , “···” represents hydrogen bonding), and boron hydroxyl group adjacent to oxygen (=BOH···O(H)X, X = Si≡ or B= )^21^. ^1^H-^11^B rotational-echo double resonance (REDOR) experiment was then conducted to identify which hydrogen species are adjacent to boron. (Fig. S20) Besides [≡SiOH···O(H)X] at 2.1 ppm and [=BOH···O(H)X] at 3.1 ppm, a ^1^H signal at 2.5 ppm was observed in ^1^H-^11^B REDOR experiment, which is assigned to silanol hydroxyl groups adjacent to boron [≡SiOH···B≡] according to previous studies^21,29^. Since most boron species are still in the zeolite framework (Fig. 4a), the presence of silanol adjacent to boron supports the existence of [B(OSi≡)_3−*x*_(OH)_*x*_] in activated B-MFI. 2D ^1^H-^1^H double-quantum (DQ) MAS NMR spectra are shown in Fig. 4d. The autocorrelation signal at (1.8, 3.6) ppm is derived from the interaction between terminal silanol hydroxyl groups^29^. The presence of the off-diagonal peak pair at (3.3, 5.4) and (2.1, 5.4) ppm indicates the spatial proximity of the silanol and boron hydroxyl groups [=BOH···O(H)Si≡] (*δ*_DQ_(5.4 ppm) = 2.1 ppm + 3.3 ppm), supporting the hydroxylation of B–O–Si linkage during the activation of B-MFI. The peak pair at (3.7, 7.0) and (3.3, 7.0) ppm is likely derived from the spatial proximity of boron hydroxyl groups. Since the boron content is B-MFI is relatively low (the molar ratio of Si/B ≈ 60), it is unlikely for two framework boron atoms to be located close to one another. Thus, this NMR spectral feature could be better attributed to [B(OSi≡)(OH)_2_], where two hydroxyl groups are bonded to the same boron atom. However, the existence of [B(OSi≡)_2_(OH)] species cannot be excluded in activated B-MFI by these experiments. The strong signals at 2.1 and 2.5 ppm in the ^1^H-^11^B REDOR spectra of activated B-MFI suggest that most boron hydroxyl groups interact extensively with adjacent silanol groups (Fig. S20), and thus the [B(OSi≡)_3−*x*_(OH)_*x*_] species could be more accurately denoted by [B(OSi≡)_3−*x*_(OH···O(H)Si≡)_*x*_] to highlight to spatial proximity of boron and silanol groups.

DFT calculations suggest that the hydroxylation of isolated boron sites is beneficial to the stabilization of potential gas-phase radicals. (Fig. 4e) Hydroperoxyl radical (HOO•) was proposed as a potential H-abstractor of alkane in oxidative dehydrogenation^11,12^. Our previous study show that treating HOO• as the active intermediate could rationalize the observed second-order dependence on *p*_C3H8_ on boron-based catalysts^12^. The binding energy of HOO• on [B(OSi≡)_3_] was calculated to be 1.61 eV (more positive values indicate less favorable binding), which gradually decreased to 0.87 and 0.49 eV as one and two B−O−Si bonds were hydrolyzed, respectively (Fig. 4e). Stabilization of HOO• on hydroxylated framework boron species could be largely attributed to the hydrogen-bonding interaction between HOO• and the hydroxyl group(s).

### ODHP on B-BEA

**Fig. 5:**
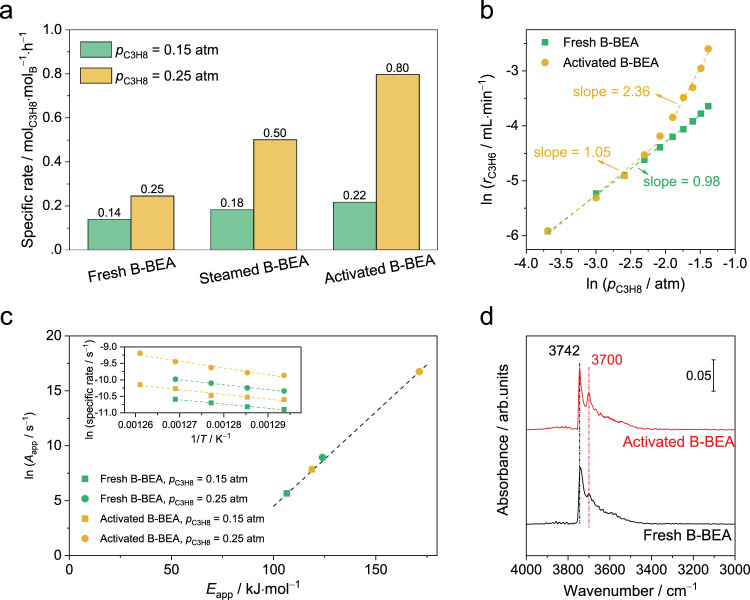
Influence of steaming on ODHP properties and hydroxylation of B-BEA. **a** Specific C_3_H_6_ formation rate of different B-BEA samples. Reaction conditions: 803 K, total gas flow = 40 mL·min^−1^, 50 mg catalyst, *p*_O2_ = 0.125 atm with balancing N_2_. **b** The reaction order of *r*_C3H6_ with respect to *p*_C3H8_ on fresh and activated B-BEA samples. Reaction conditions: 773 K, total gas flow = 40 mL·min^−1^, *p*_O2_ = 0.125 atm with balancing N_2_. **c** The compensation effect between *E*_app_ and *A*_app_. The inset are corresponding Arrhenius plots. Reaction conditions: 773 to 793 K, total gas flow = 40 mL·min^−1^, 50 mg catalyst, *p*_O2_ = 0.125 atm with balancing N_2_. **d** Transmission IR spectra of dehydrated fresh and activated B-BEA in the OH stretching vibration region under vacuum at 373 K.

To probe the generality of findings on B-MFI, effect of steaming treatment on ODHP activity and kinetics on B-BEA was investigated. XRD and N_2_ physisorption results confirm the framework type and crystallinity of the synthesized B-BEA. (Figs. S21, S22 and Table S8) ^11^B NMR spectra indicate that most boron atoms are incorporated into the zeolite framework and remain unchanged during the steaming treatment. (Figs. S23, S24 and Table S9) After 12 h of steaming treatment, the ODHP activity of fresh B-BEA increases from 0.25 to 0.50 mol_C3H6_·mol_B_^−1^·h^−1^ at *p*_C3H8_ = 0.25 atm. (Fig. 5a) Similar to B-MFI, fully steamed B-BEA can be further activated in ODHP during the induction period (Fig. S25). The apparent C_3_H_8_ order of B-BEA also increases from 0.98 to 2.36 as *p*_C3H8_ increases beyond 0.15 atm. (Fig. 5b) The measured *E*_app_ and ln (*A*_app_) also exhibits the linear correlation similar to that on B-MFI. (Fig. 5c and Table S10) Overall, the catalytic behaviors of B-BEA and B-MFI in ODHP are consistent.

Hydroxylation of boron sites also occurs during the steaming treatment on B-BEA. The conversion from B[4]-Fr to B[3]-Fr, which corresponds to the hydroxylation of isolated boron site, was also observed on activated B-BEA in ^11^B NMR spectra^34^. (Fig. S23 and Table S9) A shoulder peak at −103 ppm for [Si(OSi≡)_3_(OH)] in ^29^Si NMR spectra appears after steaming treatment, implying the hydrolysis of Si−O−X^53^. (Fig. S24) The increase of B−OH is further supported by IR spectra. (Fig. 5d) The IR peaks of isolated Si−OH (3742 cm^−1^) and isolated B−OH (3700 cm^−1^) were observed in OH stretching vibration region on B-BEA^22,27,53^. The intensity of IR peak corresponding to B−OH becomes much stronger on activated B-BEA than that on fresh B-BEA, indicating the hydrolysis of B−O−Si linkages. The similarities in the correlation between boron speciation and ODHP kinetics on B-MFI and B-BEA suggest that effect of the degree of framework boron hydroxylation on ODHP is general among isolated boron sites embedded in zeolites.

## Discussion

### Correlation among kinetic variables of ODHP on boron-based catalysts

**Fig. 6:**
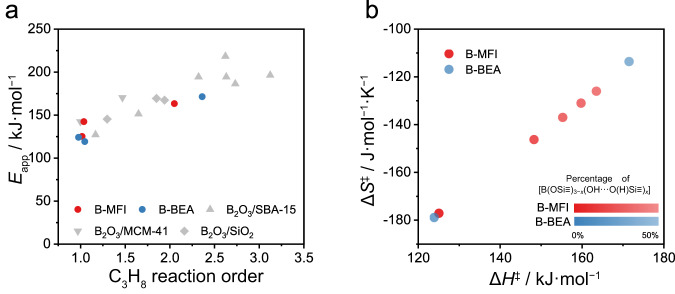
Correlation among kinetic variables in boron-catalyzing ODHP. **a** Correlation between apparent C_3_H_8_ order and *E*_app_ on different B-containing catalysts in ODHP. The data of supported B_2_O_3_ catalysts (grey symbols) comes from our previous study^27^. **b** The influence of the degree of hydroxylation on the compensation effect of activation enthalpies and entropies in ODHP on B-MFI. The color of dots represent the percentage of [B(OSi ≡)_3−*x*_(OH‧‧‧O(H)Si ≡)_*x*_] in borosilicate zeolites, quantified by the results of ^11^B NMR spectra. (Tables S7, S9).

Trends of C_3_H_8_ reaction order and *E*_app_ provide insights into ODHP mechanisms on isolated boron sites in zeolite frameworks. There is a general linear correlation between C_3_H_8_ reaction order and *E*_app_ on boron-containing catalysts in ODHP (Fig. 6a). Our recent work demonstrated that this correlation among aggregated boron species stemmed from the gradual shift from a surface-mediated mechanism to a gas-phase radical mechanism as the C_3_H_8_ reaction order increased beyond unity (grey symbols in Fig. 6a)^27^. Surface-mediated mechanisms typically lead to first-order kinetics with respect to C_3_H_8_, while gas-phase radical mechanisms could lead to C_3_H_8_ reaction orders of up to 3^27^. It should be noted that kinetic studies only probe the most populated reaction pathway, so that a first-order kinetics does not rule out the presence of radicals in the reaction system. Instead, the radical-mediated pathway is insufficiently populated to contribute significantly to the overall reactivity, potentially due to the slow production rates or fast quenching of radicals. Since B-MFI and B-BEA fit in the same trend (blue and red symbols in Fig. 6a), we propose that ODHP on isolated boron sites also have contributions from both the surface-mediated and gas-phase radical mechanisms, but with a key difference. Aggregated boron species, e.g., B_2_O_3_ and h-BN, were known to mediate gas-phase radical pathway, so the first-order kinetics for C_3_H_8_ on B_2_O_3_ nanoparticles in mesopores of SBA-15 and MCM-41 at low *p*_C3H8_ (< 0.10 atm) was attributed to the rapid quenching of radicals by colliding with the pore walls^27^. On fresh B-MFI and B-BEA, first-order kinetics for C_3_H_8_ was observed within the entire *p*_C3H8_ range investigated (0.025 atm < *p*_C3H8_ < 0.25 atm), suggesting that [B(OSi≡)_3_] likely only facilitates the surface-mediated pathway in ODHP. The pore sizes of B-MFI (0.55 nm) and B-BEA (0.67 nm) are smaller than those in MCM-41 (3.2 nm) and SBA-15 (8.7 nm), so quenching of gas-phase radicals is expected to be more effective in zeolites. Meanwhile, the difference in pore sizes among these materials is minor in comparison to the mean-free-path of gas at ODHP temperature (~150 nm)^27^. In addition, second-order kinetics for C_3_H_8_ was observed on activated B-MFI and B-BEA, confirming that the size of zeolitic pores alone cannot suppress the gas-phase radical pathway at high *p*_C3H8_. The degree of its hydroxylation of framework boron species plays a decisive role, i.e., [B(OSi≡)_3_] for the surface-mediated pathway and [B(OSi≡)_3−*x*_(OH···O(H)Si≡)_*x*_] for the gas-phase radical pathway in ODHP. (Fig. 4e) The detection of hydroxyl groups on spent h-BN and B_2_O_3_ catalysts, on which the radical mechanism is dominant in ODHP, is also consistent this hypothesis^16–18^.

**Fig. 7:**
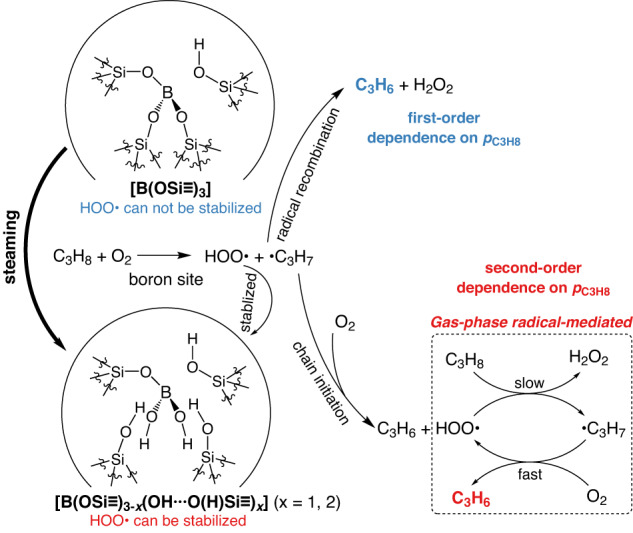
Schematic illustration of boron species transformation on B-MFI and ODHP mechanisms via different pathways. ODHP reaction pathways and C_3_H_8_ reaction order on different isolated boron sites in B-MFI are presented. C_3_H_6_ formation via surface-mediated pathway on [B(OSi ≡)_3_] is shown in the upper part and gas-phase radical pathway on [B(OSi ≡)_3−*x*_(OH···O(H)Si ≡)_*x*_] is shown in the lower part.

The highly consistent linear correlation between activation enthalpies and entropies in ODHP on B-MFI at different stages of activation (Fig. 6b and S26) indicating that the ODHP mechanism is largely independent of zeolite framework type but sensitive to the degree of hydroxylation or the coordination environment of boron. As the extent of hydroxylation in B-MFI increases from 8 to 41% (Fig. 6b), both Δ*H*^‡^ and Δ*S*^‡^ increases, suggesting increasing contributions from the gas-phase radical mechanism to the overall reactivity. The activation enthalpy-entropy correlation on B-MFI could be rationalized as changes in the relative contributions from the surface-mediated and gas-phase radical mechanisms with varying [B(OSi≡)_3_]/[B(OSi≡)_3−*x*_(OH···O(H)Si≡)_*x*_] ratios. While framework [B(OSi≡)_3_] is unique in being able to facilitate ODHP only through a mostly surface-mediated pathway, hydroxylated boron species, regardless whether in isolated or aggregated form, are able to enable both mechanisms, with the gas-phase radical mechanism being dominant at high *p*_C3H8_. Direct comparison of ODHP between boron-containing zeolites and aggregated boron species, e.g., B_2_O_3_ and h-BN, is hampered by the difficulty in determining the density of surface boron sites. When normalized by the mass loading of boron, supported B_2_O_3_/SBA-15 catalyst with a low boron loading (0.5 wt%, B_2_O_3_ basis) exhibits comparable ODHP activity with borosilicate zeolites (Fig. S27). The specific ODHP activity of h-BN is much lower, likely due to the much lower dispersion of boron. With the concomitant evolution of the degree of hydroxylation of framework boron species and kinetic variables, including C_3_H_8_ reaction order, *E*_app_ and *A*_app_ on B-MFI, it could be inferred that [B(OSi≡)_3_] is likely responsible for the surface-mediated mechanism, while [B(OSi≡)_3−*x*_(OH···O(H)Si≡)_*x*_] stabilizes key radcial species (such as HOO•) to initiate the gas-phase radical pathway, leading to a second-order kinetics with respect to C_3_H_8_ (Fig. 7).

In summary, we demonstrated that the degree of hydroxylation of isolated boron sites in zeolites has a major impact on the rate and mechanism in ODHP. Steaming treatment markedly promotes the ODHP activity of B-MFI by hydrolyzing framework [B(OSi≡)_3_] to [B(OSi≡)_3−*x*_(OH···O(H)Si≡)_*x*_]. The hydroxylated boron sites are able to facilitate the gas-phase radical reaction by stabilizing radical species, which leads to more than an order of magnitude increase in the C_3_H_6_ formation rate on B-MFI. This mechanism is supported by the shift from first- to second-order kinetics with respect to C_3_H_8_ as [B(OSi≡)_3_] is converted to [B(OSi≡)_3−*x*_(OH···O(H)Si≡)_*x*_]. Similar observations were made on B-BEA, suggesting the generality of proposed mechanism. The linear correlation between the activation enthalpy and entropy on B-MFI with different degrees of hydroxylation shows that the relative contributions of the surface-mediated and the gas-phase radical-mediated mechanisms determine the measured kinetic variables.

## Methods

### Catalysts preparation

#### Materials

Boric acid (H_3_BO_3_), 25 *wt*% tetrapropylammonium hydroxide (TPAOH) and 25 *wt*% tetraethylammonium hydroxide (TEAOH) were purchased from Alfa Aesar. Amorphous silica (SiO_2_), sodium hydroxide (NaOH) and sodium metaborate tetrahydrate (NaBO_2_·4H_2_O) were purchased from Aladdin Industrial Inc. Silicalite-1 and H-ZSM-5 samples were purchased from Tianjin Nankai University Catalyst Co., Ltd. Home-made secondary distilled water with conductivity less than 1.5 μS·cm^−1^ was used in preparation.

#### Synthesis of B-MFI zeolite

B-MFI zeolites were prepared by a solvent-free method according to the previous procedure^21^. In a typical synthesis, 0.03 g of H_3_BO_3_ and 2 g of SiO_2_ were firstly mixed in an agate mortar. Then, 2.6 g of 25 *wt*% TPAOH solution was added and the mixture was ground for 15 min. The mixture was transferred into a 10 mL Teflon autoclave and heated at 453 K for 72 h. After the thermal treatment, the obtained white powders were dried at 373 K for 12 h and finally calcined at 873 K for 4 h in flowing air. The as-prepared sample is referred to as fresh B-MFI. The steaming treatment was carried out at 823 K and *p*_H2O_ = 1.8 kPa under air flow (40 mL·min^−1^). Activated B-MFI refers to fresh or steamed B-MFI sample which was treated under ODHP condition (803 K, total gas flow = 40 mL·min^−1^, *p*_C3H8_ = 0.25 atm, *p*_O2_ = 0.125 atm with balancing N_2_) for at least 6 h to finish the induction period.

#### Synthesis of B-BEA zeolite

B-BEA zeolites with *BEA framework were prepared by a hydrothermal method. In a typical synthesis, 21.5 g of 25 *wt*% TEAOH was mixed with 11.0 g of water with stirring. Then, 0.10 g of NaOH and 0.16 g of NaBO_2_·4H_2_O were added, following by stirring for 30 min. Then 4.80 g of SiO_2_ was added into the solution and stirred for another 6 h. The mixed gel was transferred into a 100 mL Teflon autoclave and heated at 413 K for 6 days. After cooling down to room temperature, the obtained product was filtered, washed with water, dried at 373 K and finally calcined at 873 K for 4 h in flowing air. The as-prepared sample is referred to as fresh B-BEA. The steaming condition and the definition of activated B-BEA are same as B-MFI.

#### Characterization

X-ray diffraction (XRD) patterns were collected on a PANalytical X-Pert3 Powder equipped with a Cu *K*_*α*_ radiation (*λ* = 1.54056 Å). 2*θ* of X-ray diffraction pattern was recorded from 5° to 50° with a scan speed of 3°/min.

MAS ^11^B and ^29^Si solid-state NMR were performed on a Bruker AVANCE III 400 MHz spectrometer at resonance frequencies of 128.42 and 79.52 MHz for the ^11^B and ^29^Si nucleus, respectively, with a 2.5 mm MAS probe at a spinning rate of 20 kHz. The ^11^B MAS NMR spectra were collected using a single-pulse sequence with a π/12 pulse length of 3.3 μs and a recycle delay of 3 s. The ^29^Si MAS NMR spectra were collected using a single-pulse sequence with a π/12 pulse length of 0.4 μs and a recycle delay of 1 s. Before the NMR tests, the samples were dehydrated at 373 K under vacuum for 24 h. The parameters used for quantifying ^11^B NMR spectrum was obtained according to the reported method^54^. The estimated correction coefficient for the satellite transitions of tetra-coordinated boron *I*_cl_(ST, B[4]) is 1.04, and the estimated correction coefficient for the central transitions caused by quadrupolar coupling of tri-coordinated boron *I*_cl_(CT, B[3]) is 0.93. The corrected factor of measured ^11^B NMR area *I*(B[4]/B[3]) is 1.12.

The ^1^H spin-echo MAS NMR, ^1^H-^11^B symmetry-based rotational-echo double-resonance (REDOR) MAS NMR and two-dimensional (2D) ^1^H-^1^H DQ-SQ MAS NMR experiments were performed on a Bruker Avance III 600 spectrometer equipped with 14.1 T and 89 mm wide-bore magnet using 3.2 mm H-X-Y triple resonances MAS probe with the corresponding Larmor frequency of 600.13 MHz for ^1^H. Prior to the above experiments, the samples were dehydrated with a home-built vacuum line at 693 K for 12 h. The chemical shift of ^1^H NMR were referenced to adamantane at 1.74 ppm. The ^1^H spin-echo MAS NMR spectra were recorded with a π/2 pulse width of 4.0 μs, a recycle delay of 5 s, an echo time of 0.33 ms and a spinning rate of 12 kHz. The ^1^H-^11^B REDOR spectra were performed with a spinning rate of 12 kHz and a recycle delay of 5 s which accumulated 32 scans. A π/2 pulse length of 4.0 μs and π pulse length of 8.0 μs were used on the ^1^H channel with SR4 recoupling pulse. The recoupling time was 0.66 ms. The saturation pulse on the ^11^B channel was with duration of 125 μs (1.5 Tr). The ^1^H-^1^H DQ-SQ MAS NMR spectra were excited and reconverted with the POST-C7 pulse sequence with a spinning rate of 12 kHz and a recycle delay of 2 s. The increment in the indirect dimension (*t*_1_) was set to 83.34 μs, and 32 scans were acquired for each *t*_1_ increment.

Fourier transform infrared (FTIR) spectroscopy experiments were performed on a Bruker Invenio-S spectrometer in a custom transmission cell by using pyridine as the probe molecule. The samples were pressed into self-supporting wafers of about 10 mg and loaded into a sample holder for vertical alignment in the infrared beam. Spectra presented were 64 coadded scans per spectrum at a spectral resolution of 4 cm^−1^. Vacuum levels of < 0.01 mTorr in the transmission cell were achieved by connecting to a vacuum manifold equipped with a mechanical pump (Agilent) and a diffusion pump (Agilent). The transmission cell was heated by heating rings controlled by a temperature programmed PID controller. Prior to all experiments, the samples were evacuated at 823 K for 1 h to remove adsorbed molecules. The samples were then cooled down to 298 K under vacuum for further characterization.

Scanning electron microscopy (SEM) experiments were performed on a Zeiss Merlin Compact electron microscope with an acceleration voltage of 2 kV.

The element compositions of B-MFI, ZSM-5 and B-BEA zeolites were measured by inductively coupled plasma-atomic emission spectroscopy (ICP-AES) over Teledyne Leeman Labs Prodigy 7 after digestion with 2 *vol*% HF.

The N_2_ adsorption-desorption isotherms were measured at 277 K over a Micromeritics Tristar II 3030 analyzer. Before the tests, all the materials were evacuated at 363 K for 1 h and 573 K for 6 h. Brunauer-Emmett-Teller (BET) surface area are calculated from adsorption data in range of *p*/*p*_0_ = 0.05–0.30. The micropore evaluation was used t-plot method based on Harkins-Jura model. The linearization of t-plot is in range from thickness = 3.5–5 Å, corresponding to *p*/*p*_0_ = 0.10–0.27.

#### Computational method

The unit cell of B-MFI is obtained from the peak positions in XRD patterns with the cell parameters of *a* = 20.07 Å, *b* = 19.88 Å, *c* = 13.39 Å, *α* = *β* = *γ* = 90°. DFT calculations using periodic boundary condition (PBC) models are carried out with the Quickstep module of the CP2K (version 7.1) package^55^. The Perdew-Burke-Ernzerhof (PBE)^56^ functional is used in combination with Godecker-Teter-Hutter (GTH) pseudopotentials^57^ and the molecularly optimized double-*ζ* polarization quality Gaussian basis sets (DZVP-MOLOPT-SR-GTH)^58^. An 800 Ry energy cutoff, a 70 Ry relative energy cut-off, and 4 grid levels are employed. The binding energy of hydroperoxyl radical (HOO•) on isolated boron sites with different degree of hydroxylation was calculated as

1 $${E}_{{{{{{\rm{bind}}}}}}}({{{{{\rm{HOO}}}}}}\cdot )={{E}}_{{{{{{\rm{B}}}}}}-{{{{{\rm{MFI}}}}}}-{{{{{\rm{OOH}}}}}}}-{E}_{{{{{{\rm{B}}}}}}-{{{{{\rm{MFI}}}}}}}-{E}_{{{{{{{\rm{O}}}}}}}_{2}}-{\mu }_{{{{{{\rm{H}}}}}}}$$

where *E*_B-MFI-OOH_, *E*_B-MFI_, *E*_O2_ and *μ*_H_ refer to the calculated energy of B-MFI-OOH intermediate, the isolated boron sites, O_2_ in gas phase and the chemical potential of H which referenced from water formation 1/2H_2_ + 1/4O_2_ = 1/2H_2_O (*μ*_H_ = 1/2*E*_H2O_ – 1/4*E*_O2_). According to the definition of *E*_bind_(HOO•), more positive values indicate less favorable binding between B-MFI and HOO•.

#### Catalytic testing

Catalytic properties of ODHP were evaluated in a quartz fixed-bed tubular reactor with an inner diameter of 6 mm under atmospheric pressure. In a typical experiment, 200 mg mixed sample and SiC with 20 to 40 mesh were used in tests of catalytic performance with a mass ratio of 1:3. The catalyst bed was supported by 1 cm quartz wool lug. During the steaming treatment, water vapor was introduced into the reactor by gas bubbler with the *p*_H2O_ = 1.8 kPa. The reactor effluent was analyzed online by an Agilent 8890 gas chromatograph (GC) equipped with a TCD and an FID detector. O_2_, CO, CO_2_ and N_2_ were separated by 5 A column (Agilent J&W) and quantified by TCD. Hydrocarbons (C_2_H_6_, C_3_H_8_, C_2_H_4_, C_3_H_6_, CH_4_) were separated by HP-PLOT Al_2_O_3_ column (Agilent J&W) and quantified by FID. The C_3_H_8_ conversion was determined by Eq. (2).

2 $${C}_{3}{H}_{8}\,conversion=\frac{{F}_{{{{{{\rm{C}}}}}}3{{{{{\rm{H}}}}}}8,{{{{{\rm{inlet}}}}}}}-{F}_{{{{{{\rm{C}}}}}}3{{{{{\rm{H}}}}}}8,{{{{{\rm{outlet}}}}}}}}{{F}_{{{{{{\rm{C}}}}}}3{{{{{\rm{H}}}}}}8,{{{{{\rm{inlet}}}}}}}}\times 100\%$$

*F* is the volumetric flow rate under standard temperature and pressure (STP). The specific rate of C_3_H_6_ formation was determined by Eq. (3).

3 $$Specific\,rate\,of\,{C}_{{3}}{H}_{{6}}\,formation=\frac{{L}_{{{{{{\rm{C}}}}}}3{{{{{\rm{H}}}}}}6,{{{{{\rm{outlet}}}}}}}}{{n}_{{{{{{\rm{boron}}}}}}}}$$

*L* is the molar flow rate under STP and *n*_boron_ is the molar amount of boron in the measured catalyst, which is determined by ICP-AES. The selectivity of product *i* was determined by Eq. (4).

4 $$Selectivity=\frac{{n}_{i}\times {F}_{i,{{{{{\rm{outlet}}}}}}}}{3\times ({F}_{{{{{{\rm{C}}}}}}3{{{{{\rm{H}}}}}}8,\,{{{{{\rm{in}}}}}}}-{F}_{{{{{{\rm{C}}}}}}3{{{{{\rm{H}}}}}}8,\,{{{{{\rm{out}}}}}}})}\times 100\%$$

where *i* represents the products C_3_H_6_, C_2_H_4_, CH_4_, CO and CO_2_ in the effluent gas, *n*_*i*_ is the number of carbon atoms of component *i*, and *F*_*i*_ is the volumetric flow rate under STP. The carbon balance (Eq. (5)) in all experiments was higher than 98%,

5 $$Carbon\,balance=\frac{3\times {F}_{{{{{{\rm{C}}}}}}3{{{{{\rm{H}}}}}}8,{{{{{\rm{outlet}}}}}}}+\sum ({n}_{i}\times {F}_{{i},\,{{{{{\rm{outlet}}}}}}})}{3\times {F}_{{{{{{\rm{C}}}}}}3{{{{{\rm{H}}}}}}8,\,{{{{{\rm{inlet}}}}}}}}\times 100\%$$

The deactivation of catalysts was less than 5% in all cases.

### Supplementary information

Supplementary Information

Peer Review File

### Source data

Source Data

## Data Availability

The authors declare that all the relevant data within this paper and its Supplementary Information file are available from the corresponding author upon a reasonable request. Source data are provided with this paper.
