# Supplementary material for: Rationalizing kinetic behaviors of isolated boron sites catalyzed oxidative dehydrogenation of propane
Source: Nat Commun. 2023 Oct 16;14:6520. doi: 10.1038/s41467-023-42403-2 (PMC10579386; doi:10.1038/s41467-023-42403-2)
Supplement: Supplementary file 1 — Supplementary Information [file 41467_2023_42403_MOESM1_ESM.pdf]

Supplementary Information

Rationalizing Kinetic Behaviors of Isolated Boron Sites Catalyzed Oxidative Dehydrogenation of Propane

Hao Tian[1,2], Wenying Li[3], Linhai He[4], Yunzhu Zhong[1,2], Shutao Xu[4], Hai Xiao[3], Bingjun Xu[1,2]*

[1]*College of Chemistry and Molecular Engineering, Peking University, Beijing 100871, China*

[2] *Beijing National Laboratory for Molecular Sciences, Beijing 100871, China*

[3]*Department of Chemistry and Key Laboratory of Organic Optoelectronics & Molecular Engineering of Ministry of Education, Tsinghua University, Beijing 100084, China*

[4]*National Engineering Laboratory for Methanol to Olefins, Dalian National Laboratory for Clean Energy, iChEM (Collaborative Innovation Center of Chemistry for Energy Materials), Dalian Institute of Chemical Physics, Chinese Academy of Sciences, Dalian, 116023, China*

Email: b_xu@pku.edu.cn

Contents

Supplementary Figures and Tables

Fig. S1. SEM images of fresh B-MFI sample......**4**

Fig. S2. XRD patterns of fresh B-MFI and other MFI-type zeolites......**5**

Fig. S3. N_2 physisorption of fresh B-MFI......**6**

Fig. S4. ^{11}B MAS NMR spectrum of fresh B-MFI......**7**

Fig. S5. ^{29}Si NMR spectrum of fresh B-MFI......**8**

Fig. S6. Transmission IR spectra of dehydrated fresh B-MFI sample under vacuum at different temperatures......**9**

Fig. S7. The influence of boron content in fresh B-MFI on C_3H_6 formation rate in ODHP......**10**

Fig. S8. Influence of steaming on apparent C_3H_8 reaction order......**11**

Fig. S9. Induction period of fully steamed B-MFI in ODHP......**12**

Fig. S10. The influence of SiC diluent on ODHP activity......**13**

Fig. S11. B/Si molar ratio of fresh and activated B-MFI analyzed by ICP-AES......**14**

Fig. S12. SEM images of activated B-MFI......**15**

Fig. S13. XRD patterns of B-MFI samples after steaming treatment......**16**

Fig. S14. ^{29}Si NMR spectra of B-MFI samples after steaming treatment......**17**

Fig. S15. The distribution of boron species on fresh, steamed and activated B-MFI from peak fitting of ^{11}B NMR spectra......**18**

Fig. S16. Transmission IR spectra of dehydrated activated B-MFI sample under vacuum collected at different temperatures......**19**

Fig. S17. Transmission IR bands of (a) OH group, (b) tetrahedral framework boron and (c) trigonal

framework boron regions on dehydrated activated B-MFI collected at different temperatures......**20**

Fig. S18. Schematic illustration of boron species transformation in B-MFI **21**

Fig. S19. ^{1}H MAS NMR spectrum of activated B-MFI..**22**

Fig. S20. ^{1}H-^{11}B REDOR spectra of activated B-MFI..**23**

Fig. S21. N_2 physisorption of fresh B-BEA...**24**

Fig. S22. XRD patterns of fresh and activated B-BEA samples..**25**

Fig. S23. ^{11}B NMR spectra of fresh and activated B-BEA samples **26**

Fig. S24. ^{29}Si NMR spectra of fresh and activated B-BEA samples...**27**

Fig. S25. The induction period of B-BEA sample after steaming **28**

Fig. S26. Arrhenius plots of B-MFI with different degree of hydroxylation..................................**29**

Fig. S27. Specific C_3H_6 formation rates on different boron-based catalysts **30**

Table S1. The summary of kinetic properties of boron-based catalysts in oxidative dehydrogenation of propane. **31**

Table S2. Crystallographic data of B-MFI and ZSM-5 ....**33**

Table S3. Morphological properties of MFI-type zeolites..**34**

Table S4 ODHP performance of silicalite-1 ....**35**

Table S5. E_{app} and A_{app} of fresh and activated B-MFI samples **36**

Table S6. Crystallographic data of steamed and activated B-MFI samples **37**

Table S7. ^{11}B NMR peak fitting results of fresh, steamed and activated B-MFI zeolites **38**

Table S8. Morphological properties of fresh B-BEA **39**

Table S9. ^{11}B NMR peak fitting results of fresh and activated B-BEA zeolites.............................**40**

Table S10. E_{app} and A_{app} of fresh and activated B-BEA samples..**41**

Supplementary References **42**

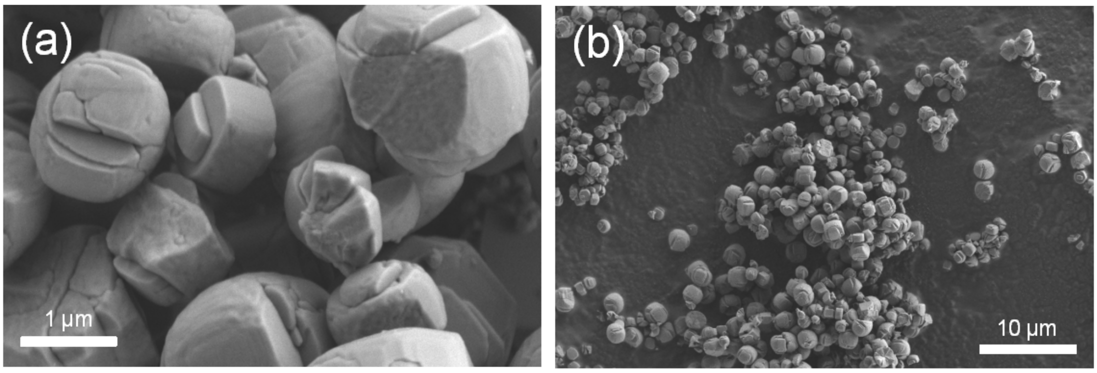

Fig. S1. SEM images of fresh B-MFI sample. (a) The morphology of fresh B-MFI particles. (b) The SEM image to present the particle size distribution of fresh B-MFI.

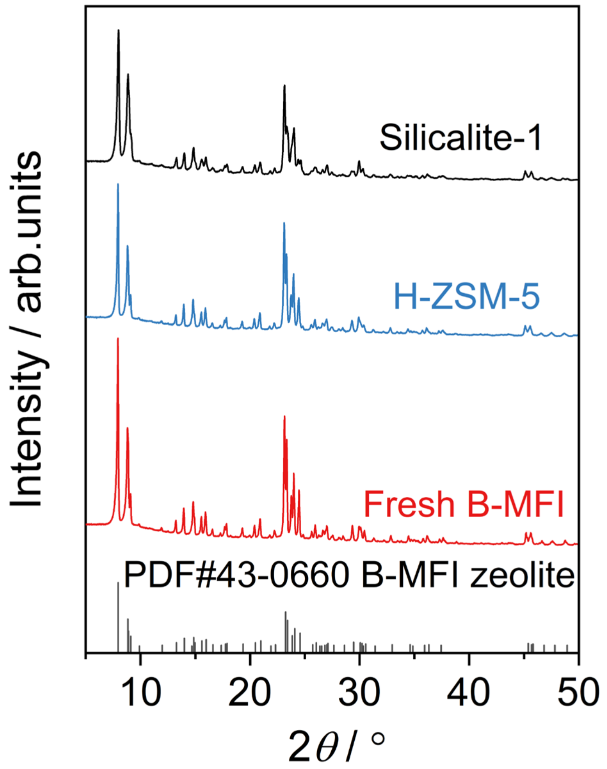

Fig. S2. XRD patterns of fresh B-MFI and other MFI-type zeolites.

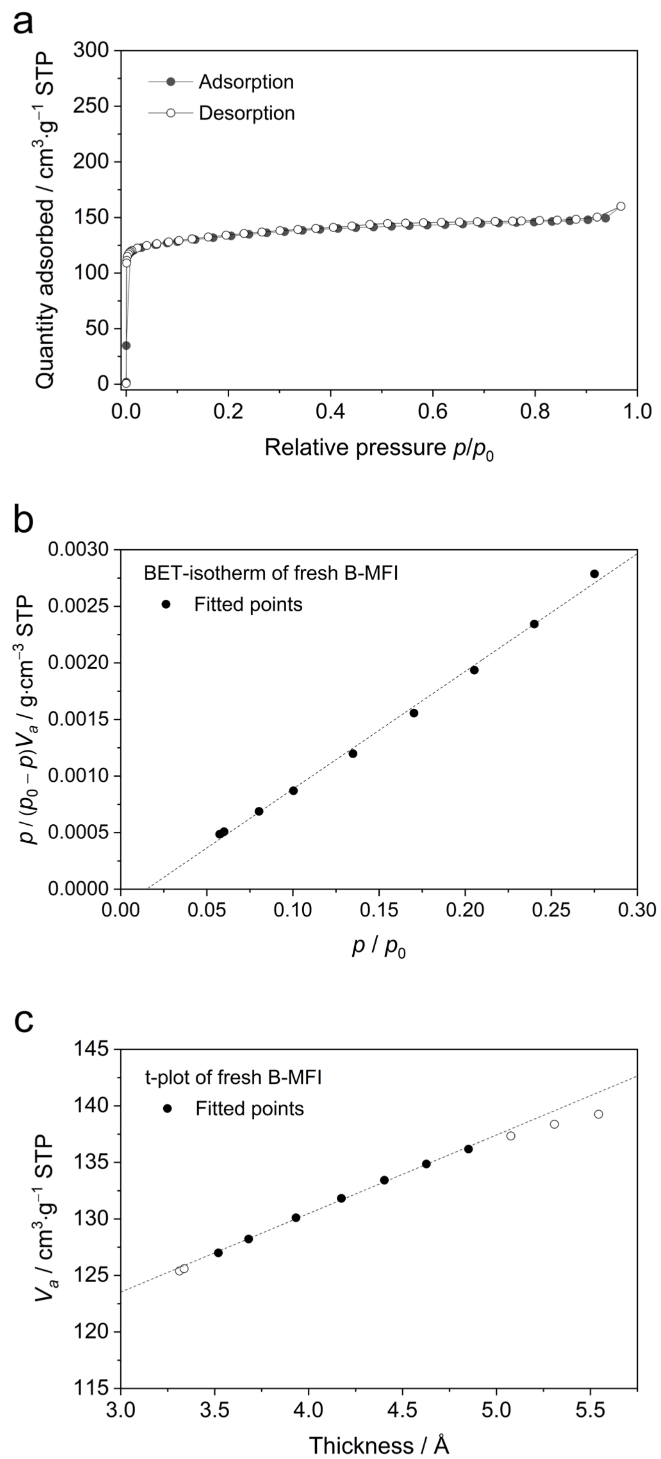

Fig. S3. N_2 physisorption of fresh B-MFI. (a) N_2 adsorption-desorption isotherm, (b) BET isotherm and (c) t-plot of fresh B-MFI sample.

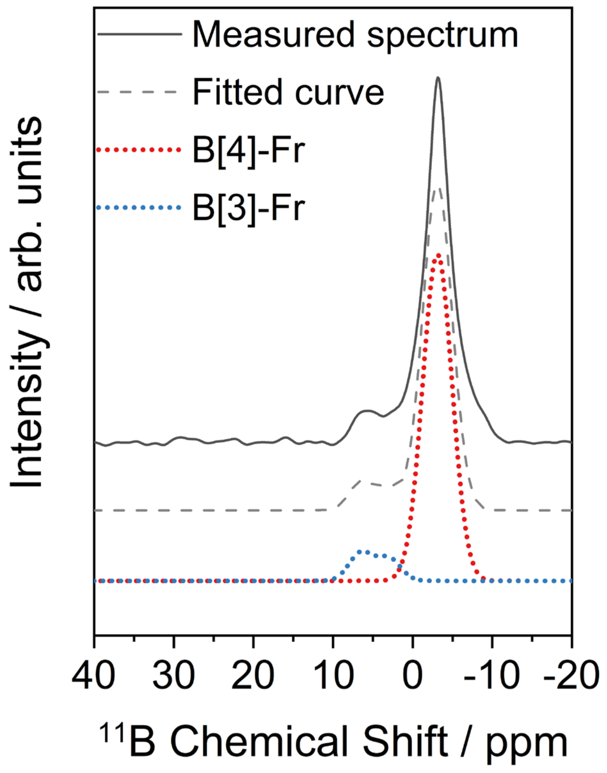

Fig. S4. ^{11}B MAS NMR spectrum of fresh B-MFI.

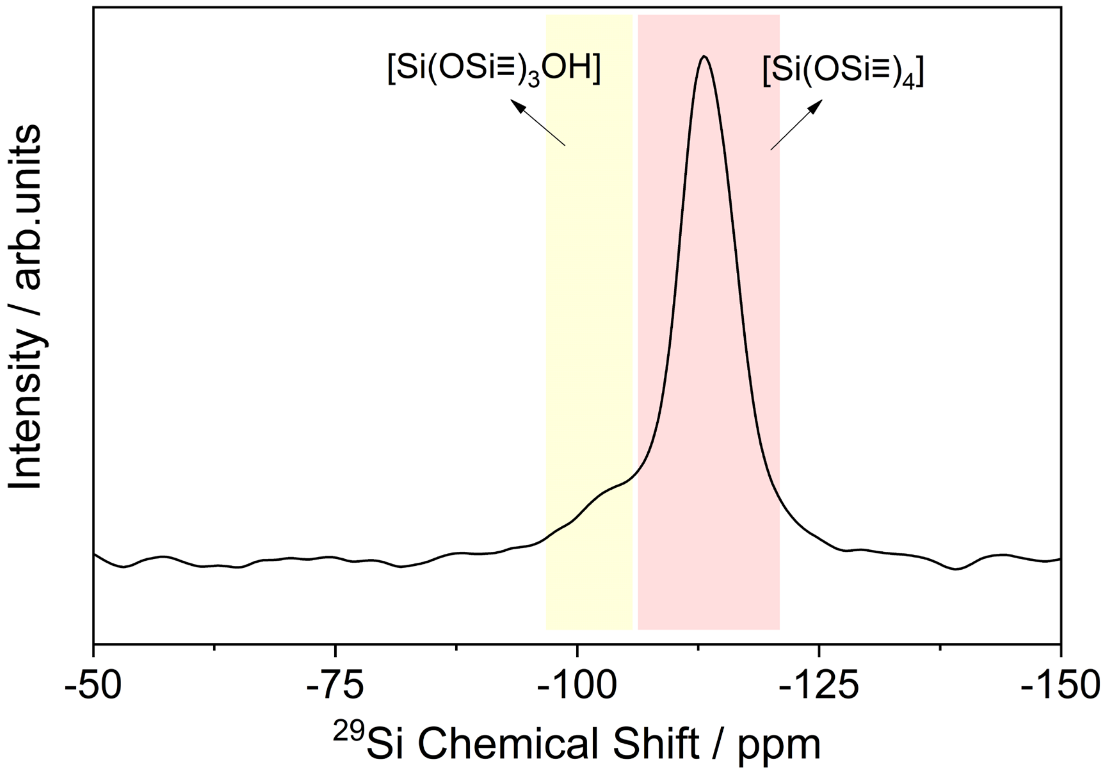

Fig. S5. ^{29}Si NMR spectrum of fresh B-MFI.

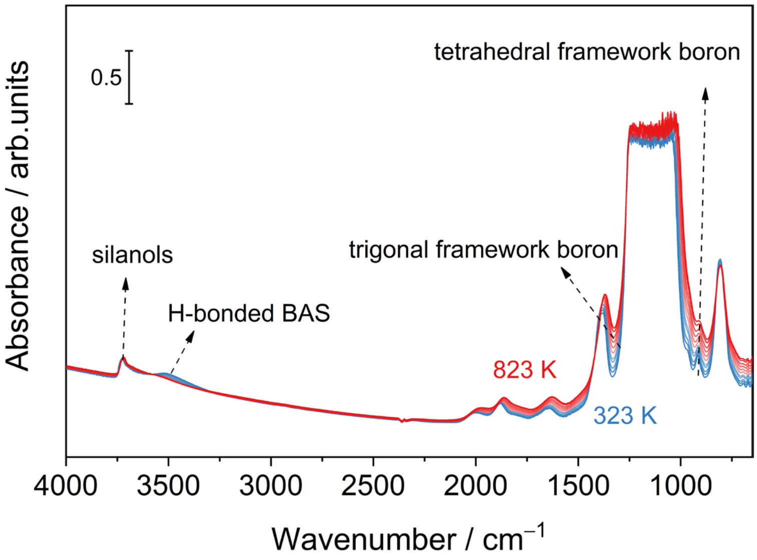

Fig. S6. Transmission IR spectra of dehydrated fresh B-MFI sample under vacuum at different temperatures.

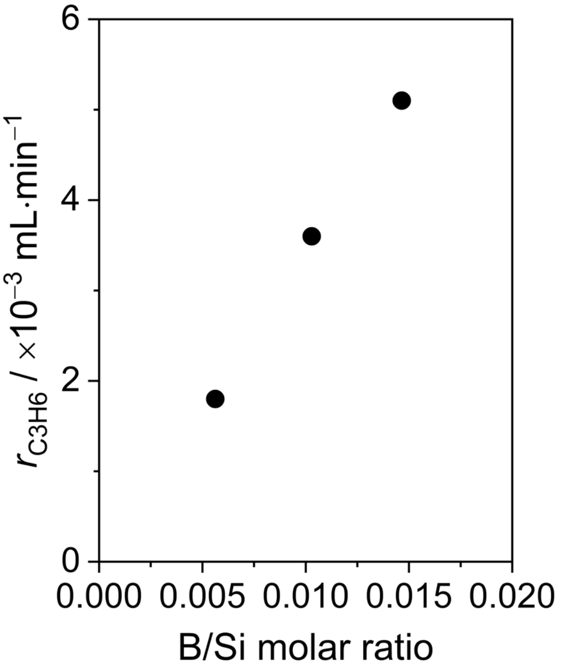

Fig. S7. The influence of boron content in fresh B-MFI on C_3H_6 formation rate in ODHP. Reaction conditions: 773 K, total gas flow = 40 mL·min^{-1}, p_{C3H8} = 0.25 atm, p_{O2} = 0.125 atm with balancing N_2.

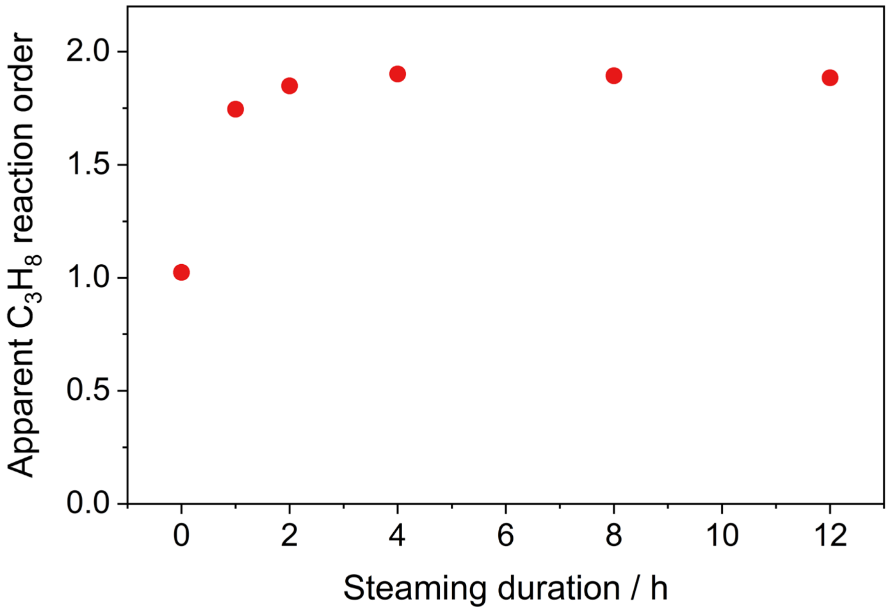

Fig. S8. Influence of steaming on apparent C_3H_8 reaction order. Reaction condition: 803 K, total gas flow = 40 mL·min^{-1}, 50 mg catalyst, p_{O2} = 0.125 atm with balancing N_2.

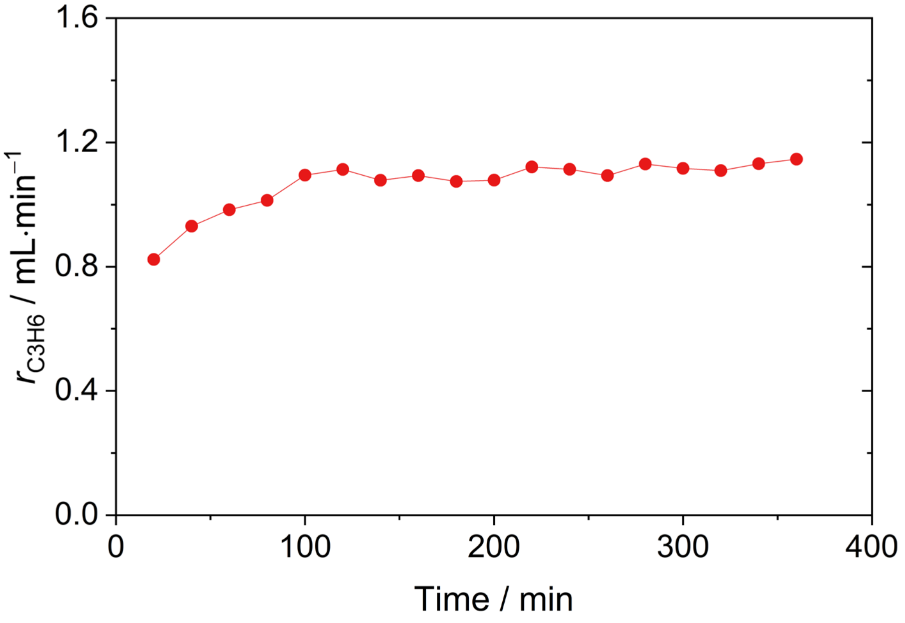

Fig. S9. Induction period of fully steamed B-MFI in ODHP. Reaction condition: 803 K, total gas flow = 40 mL·min^{-1}, p_{C3H8} = 0.25 atm, p_{O2} = 0.125 atm with balancing N_2.

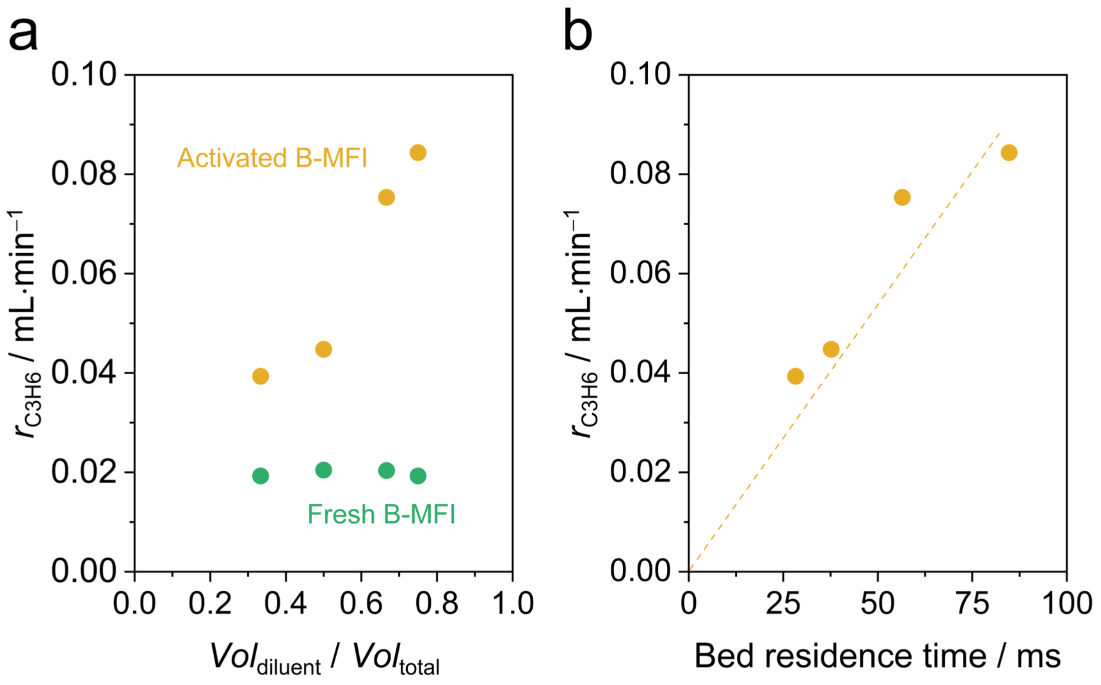

Fig. S10. The influence of SiC diluent on ODHP activity. (a) C_3H_6 formation rate of fresh and activated B-MFI at different volumetric ratios of SiC to (SiC + B-MFI). (b) Correlation between bed residence time and C_3H_6 formation rate of activated B-MFI. Reaction condition: 793 K, p_{C3H8} = 0.125 atm, p_{O2} = 0.0625 atm with balancing N_2.

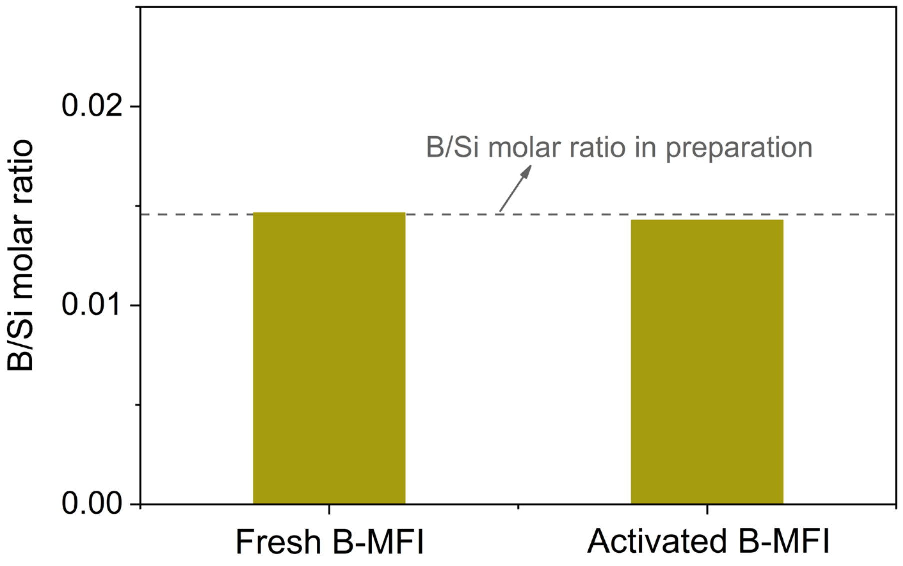

Fig. S11. B/Si molar ratio of fresh and activated B-MFI analyzed by ICP-AES.

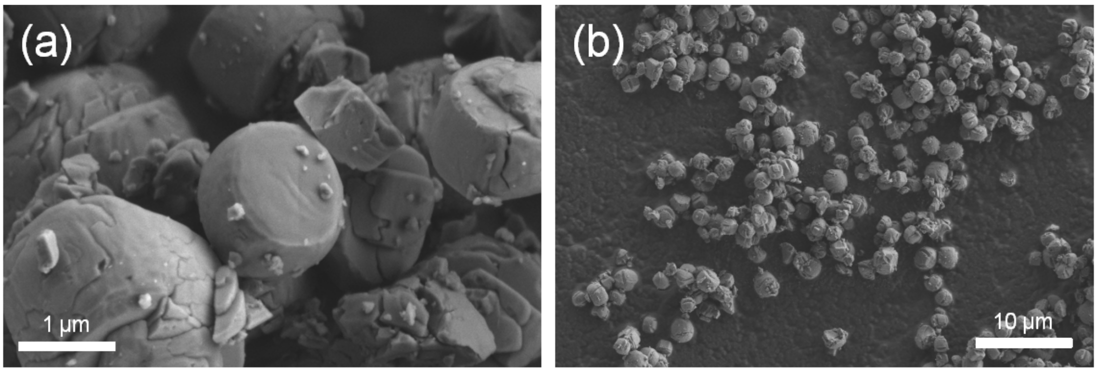

Fig. S12. SEM images of activated B-MFI. (a) The morphology of activated B-MFI particles. (b) The SEM image to present the particle size distribution of activated B-MFI.

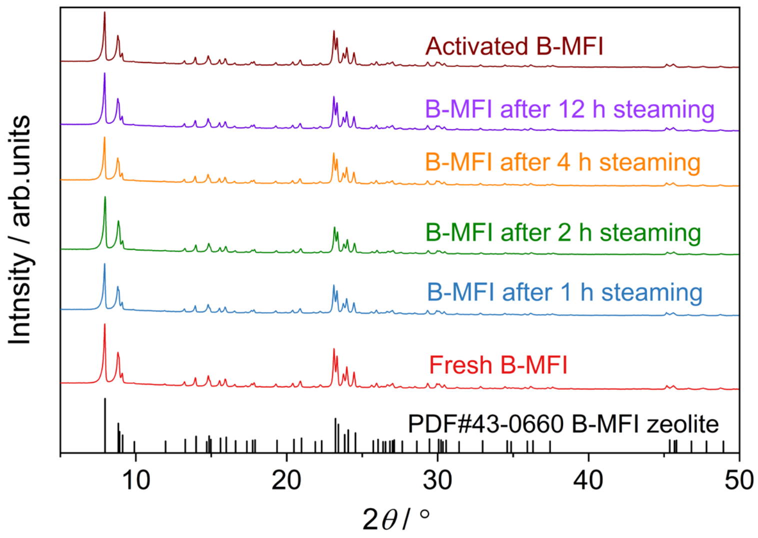

Fig. S13. XRD patterns of B-MFI samples after steaming treatment.

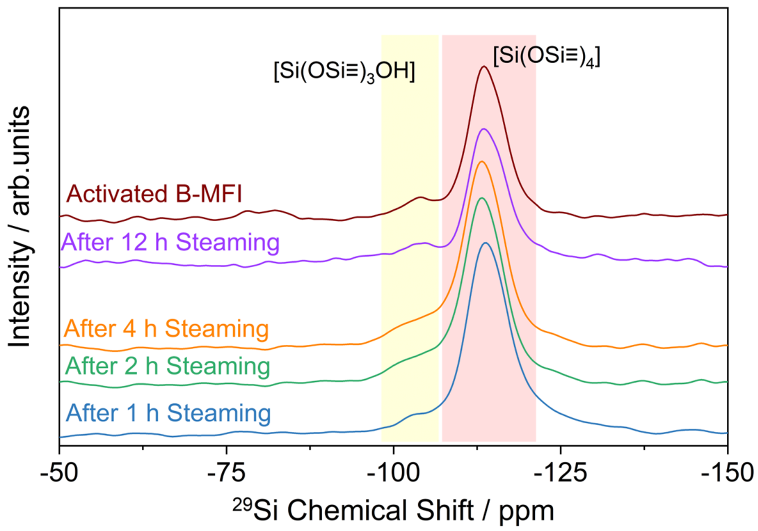

Fig. S14. ^{29}Si NMR spectra of B-MFI samples after steaming treatment.

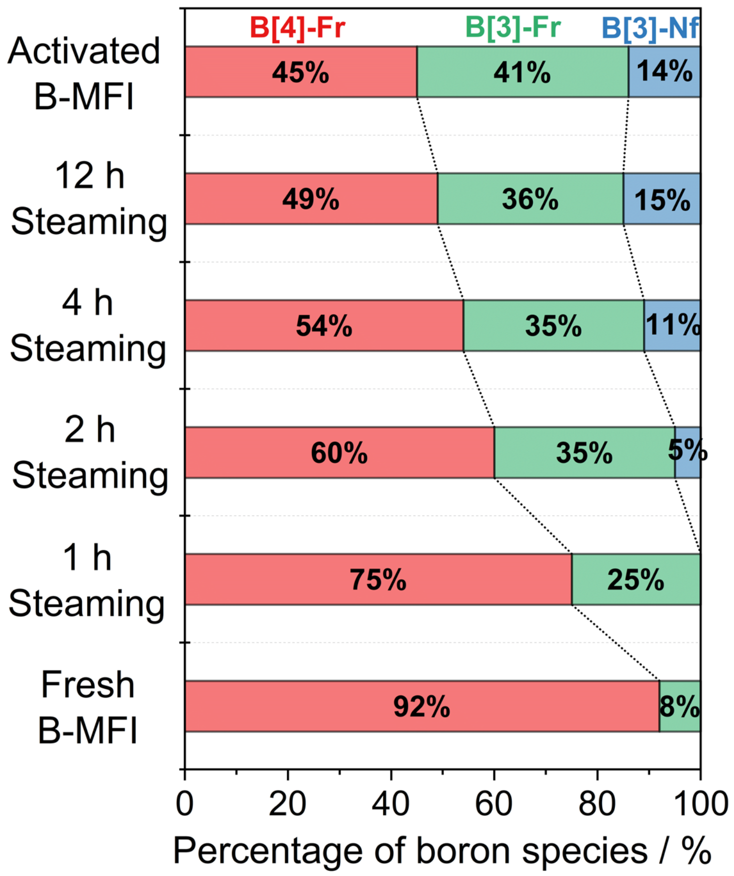

Fig. S15. The distribution of boron species on fresh, steamed and activated B-MFI from peak fitting of ^{11}B NMR spectra.

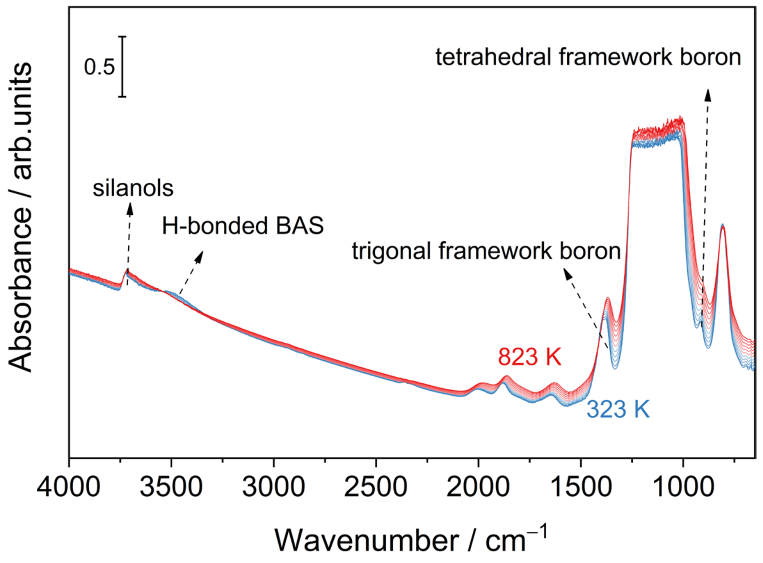

Fig. S16. Transmission IR spectra of dehydrated activated B-MFI sample under vacuum collected at different temperatures.

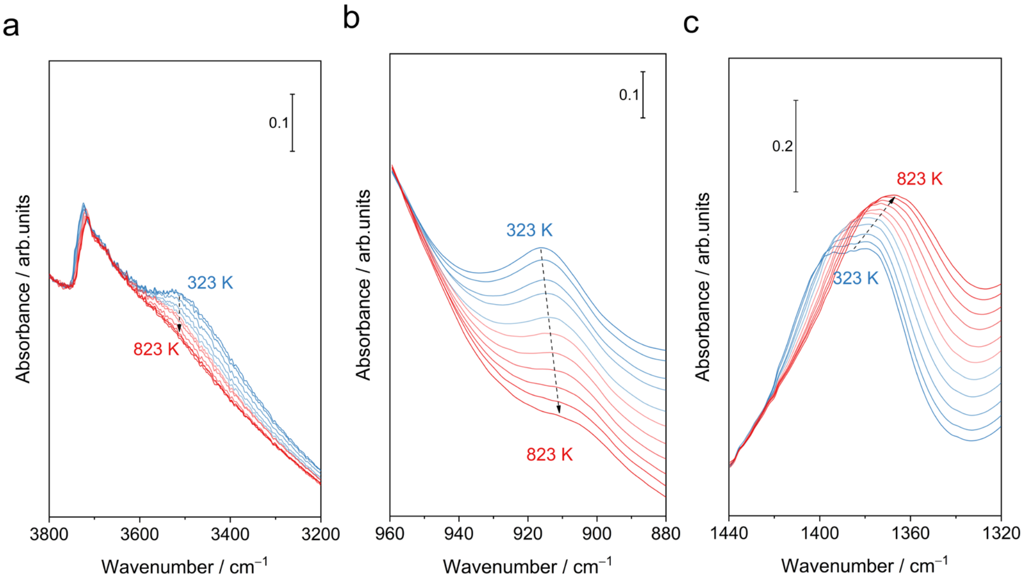

Fig. S17. Transmission IR bands of (a) OH group, (b) tetrahedral framework boron and (c) trigonal framework boron regions on dehydrated activated B-MFI collected at different temperatures.

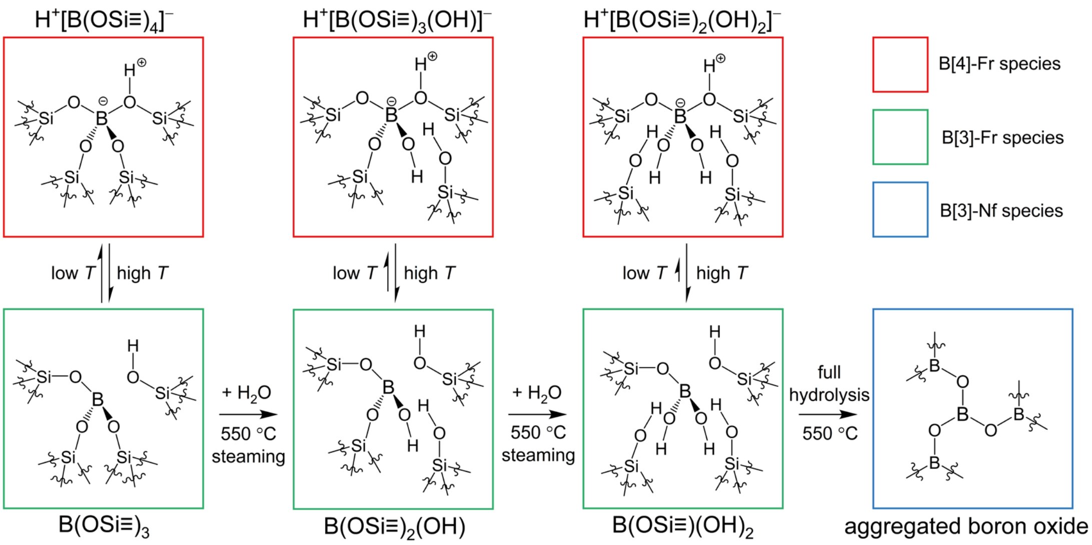

Fig. S18. Schematic illustration of boron species transformation in B-MFI.

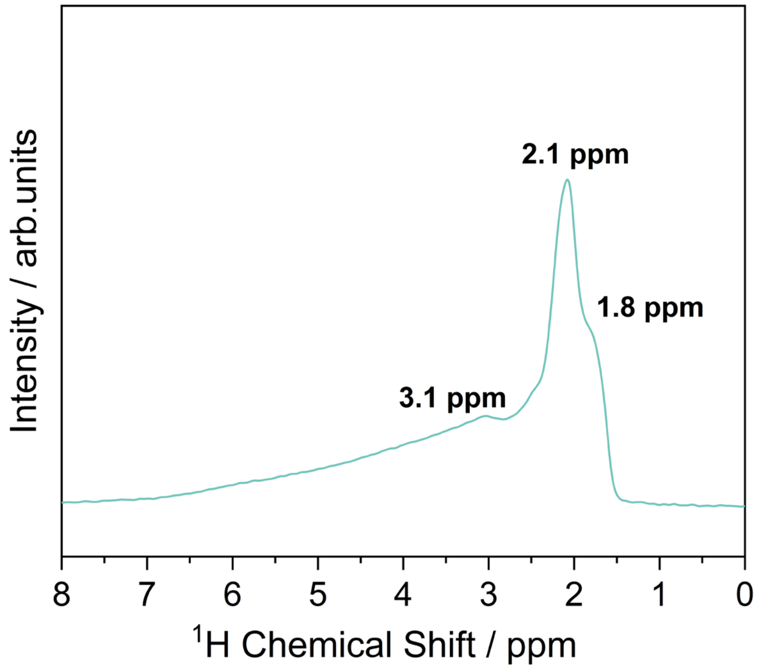

Fig. S19. ^{1}H MAS NMR spectrum of activated B-MFI.

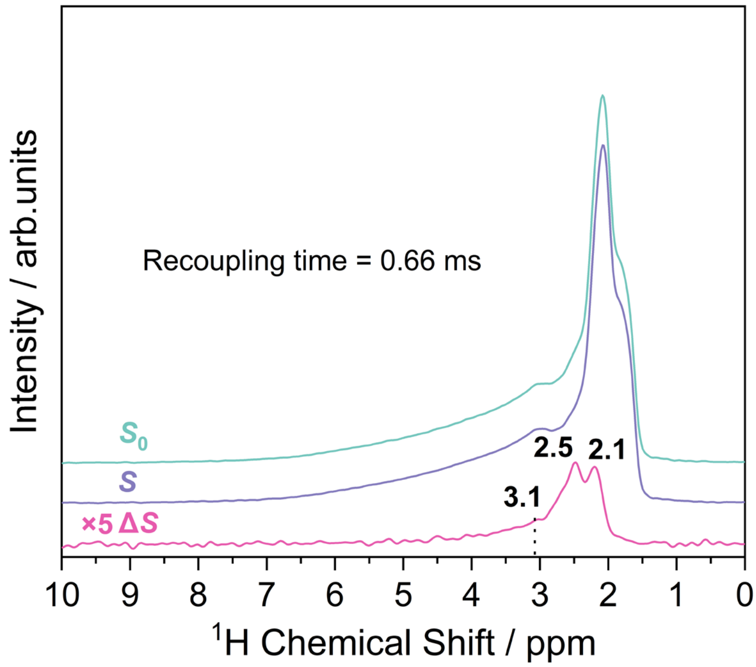

Fig. S20. ^{1}H-^{11}B REDOR spectra of activated B-MFI, combining the reference ^{1}H spectrum (S_0), ^{1}H spectrum with the application of 180° pulse trains on the ^{11}B channel (S) and the difference spectrum ($\Delta S = S_0 - S$).

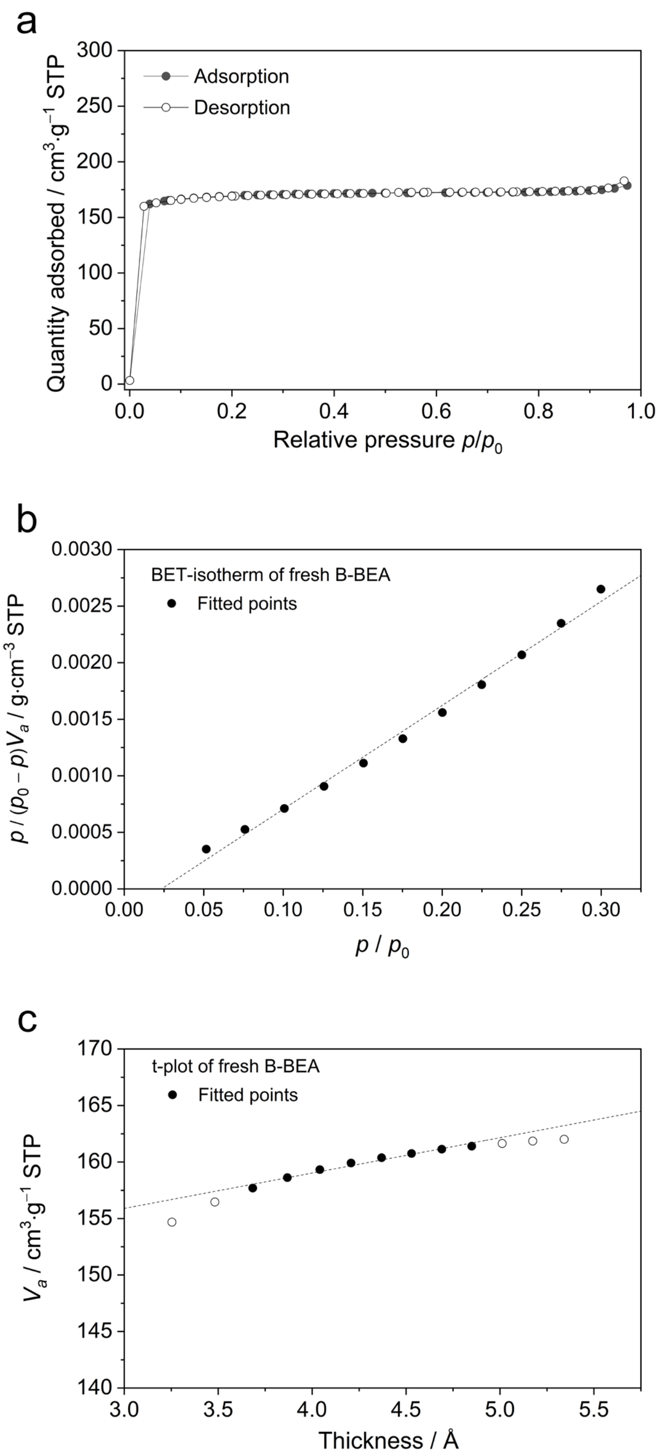

Fig. S21. N_2 physisorption of fresh B-BEA (a) N_2 adsorption-desorption isotherm, (b) BET isotherm and (c) t-plot of fresh B-BEA sample.

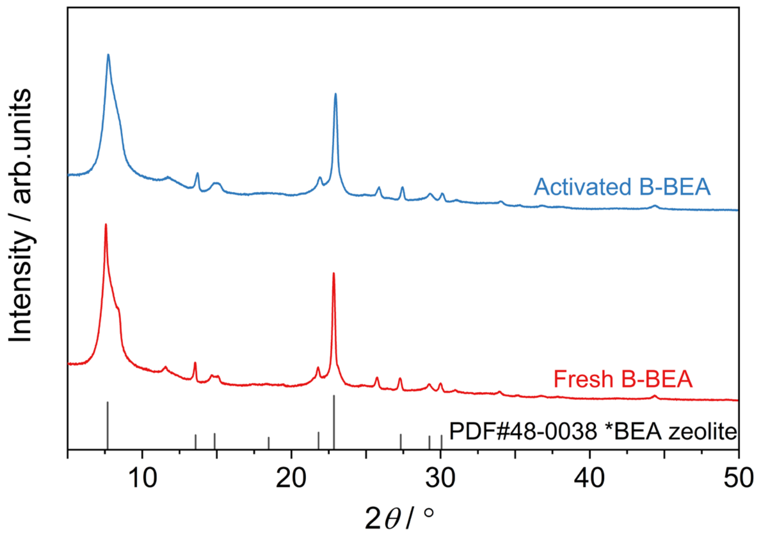

Fig. S22. XRD patterns of fresh and activated B-BEA samples.

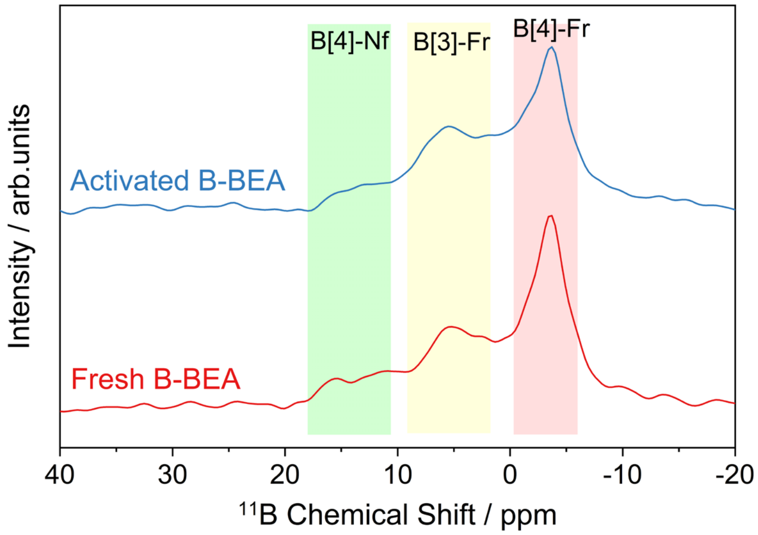

Fig. S23. ^{11}B NMR spectra of fresh and activated B-BEA samples.

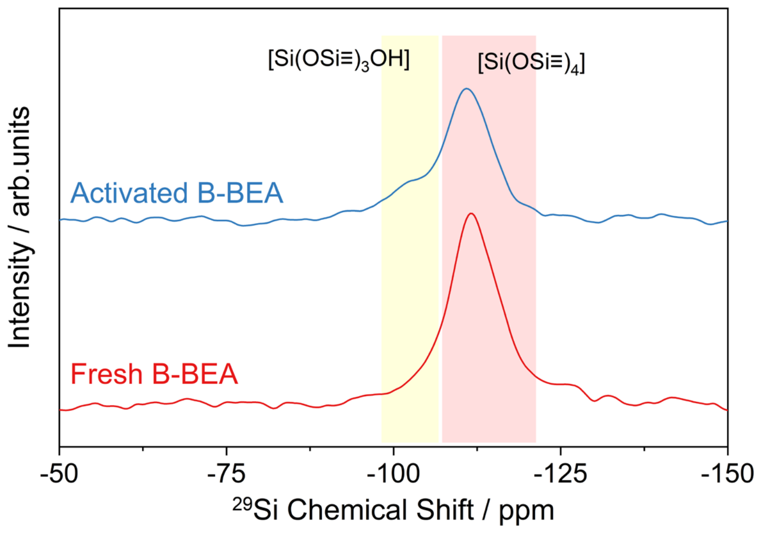

Fig. S24. ^{29}Si NMR spectra of fresh and activated B-BEA samples.

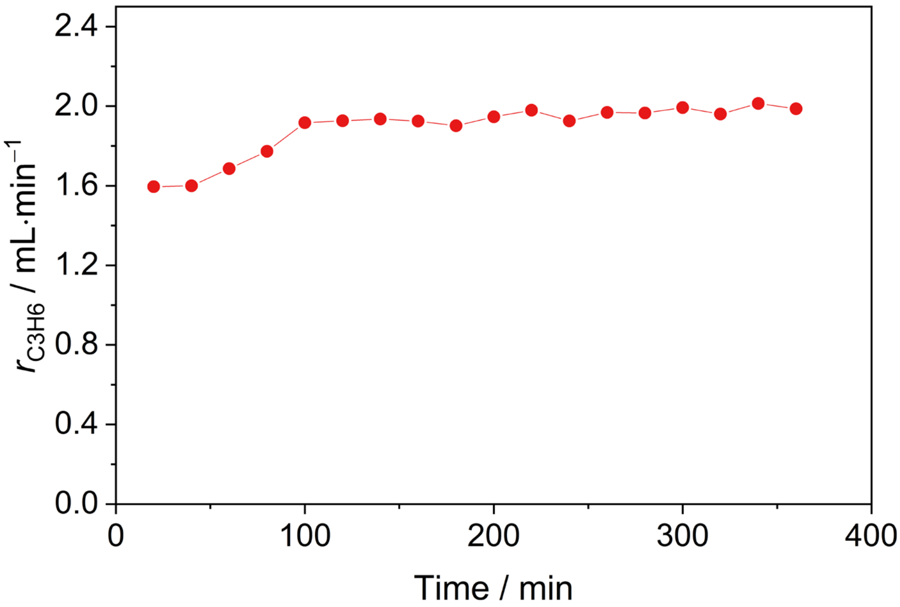

Fig. S25. The induction period of B-BEA sample after steaming. Reaction condition: 823 K, total gas flow = 40 mL·min^{-1}, p_{C3H8} = 0.25 atm, p_{O2} = 0.125 atm with balancing N_2.

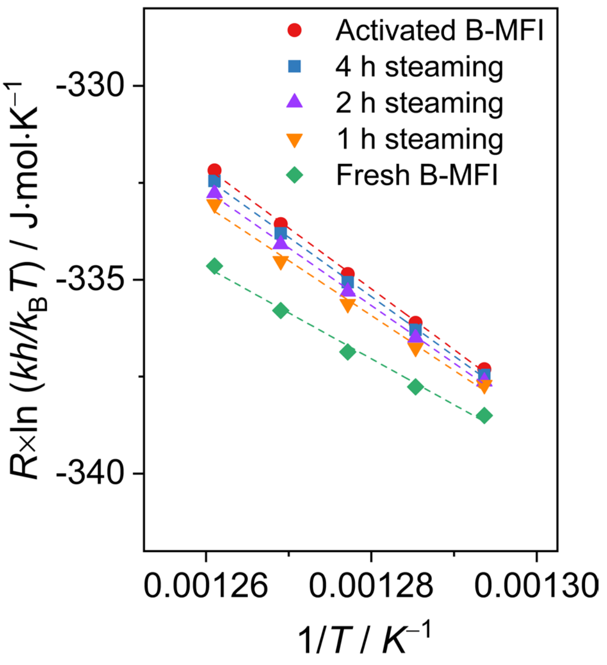

Fig. S26. Arrhenius plots of B-MFI with different degree of hydroxylation. Reaction condition: 773 K to 793 K, p_{C3H8} = 0.25 atm, p_{O2} = 0.125 atm with balancing N_2.

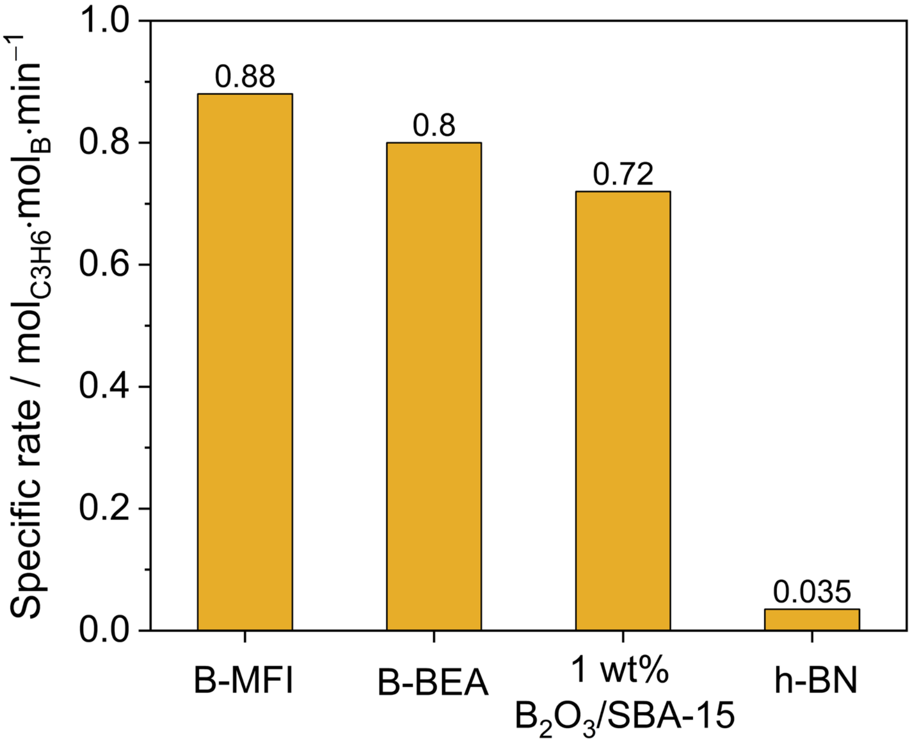

Fig. S27. Specific C_3H_6 formation rates on different boron-based catalysts. Reaction condition: T = 803 K, p_{C3H8} = 0.25 atm, p_{O2} = 0.125 atm with N_2 as balance, total gas flow = 40 mL·min^{-1}.

Table S1. The summary of kinetic properties of boron-based catalysts in oxidative dehydrogenation of propane.

Catalyst	Reaction condition	C_3H_8 order	O_2 order	E_{app} / kJ·mol^{-1}	Reference
		h-BN related catalysts			
h-BN	733 K to 763 K	2	1→0	253	[S1]
h-BN	773 K to 813 K	n.m.[1]	n.m.	213	[S2]
h-BN	793 K to 823 K	n.m.	n.m.	192.3	[S3]
h-BN	798 K	2.1	n.m.	n.m.	[S4]
h-BN	798 K, with 10% H_2O	2.2	n.m.	n.m.	[S4]
h-BN	793 K to 873 K	n.m.	n.m.	241.9 to 264.4	[S5]
h-BN	773 K to 813 K	n.m.	n.m.	239	[S6]
h-BN	733 K to 773 K	1.915	n.m.	154.4	[S7]
BNOH	773 K to 813 K	2	1→0	184	[S2]
		Supported B_2O_3 catalysts			
B_2O_3/SBA-15	678 K to 718 K	2	1→0	145	[S8]
B_2O_3/SBA-15	733 K to 753 K	1.17 to 3.12	1→0	127 to 218	[S9]
B_2O_3/SiO_2	773 K to 833 K	n.m.	n.m.	170.4	[S5]
B_2O_3/SiO_2	743 K to 773 K	1.950	n.m.	160.0	[S7]
B_2O_3/SiO_2	733 K to 753 K	1.30 to 1.94	1→0	145 to 169	[S9]
B_2O_3/SiO_2	743 K to 773 K	2	1→0	n.d.	[S10]
B_2O_3/silicate-1	783 K to 823 K	1.864	n.m.	166.7	[S7]
B_2O_3/MCM-41	733 K to 793 K	1.00 to 1.47	1→0	142 to 170	[S9]

Other boron-based catalysts					
Silicon boride	773 K to 803 K	2.2	1→0	244	[S11]
BPO_4	758 K to 813 K	n.m.	0.5	200 to 207	[S12]
Borosilicate zeolites					
B-MFI	703 K to 753 K	0.984	1→0	80.8	[S7]
B-BEA	693 K to 773 K	1	n.m.	83.7	[S7]
B-MWW	743 K to 793 K	2	0.5	204 to 212	[S13]

[1]n.m.: not measured.

Table S2. Crystallographic data of B-MFI and ZSM-5. The mass loadings and B or Al atoms per unit cell are calculated from the results of ICP-AES. The lattice constants and unit cell volumes are calculated from the peak positions in XRD patterns based on the orthorhombic unit cell.

Sample	B / wt%	B/unit cell	Al / wt%	Al/unit cell	*a* / Å	*b* / Å	*c* / Å	V_{cell} / Å^3
B-MFI	0.18	1.0	-	-	20.07	19.88	13.39	5342
H-ZSM-5	-	-	0.88	1.9	20.08	20.08	13.35	5383
Silicalite-1	-	-	-	-	20.06	19.95	13.40	5363

Table S3. Morphological properties of MFI-type zeolites.

Sample	Surface area / $m^2 \cdot g^{-1}$	Pore volume / $cm^3 \cdot g^{-1}$	Micropore area / $m^2 \cdot g^{-1}$	Micropore volume / $m^2 \cdot g^{-1}$
B-MFI	412	0.23	313	0.14
H-ZSM-5	328	0.25	238	0.13
Silicalite-1	321	0.26	244	0.13

Table S4. ODHP performance of silicalite-1. Reaction condition: 773 K, 500 mg catalyst, total gas flow = 40 mL·min−1, p_{C3H8} = 0.25 atm, p_{O2} = 0.125 atm with balancing N_2.

C_3H_8 conversion / %	C_3H_6 selectivity / %	CO selectivity / %	CO_2 selectivity / %
<0.1	0	72	28

Table S5. E_{app} and A_{app} of fresh and activated B-MFI samples.

Sample	p_{C3H8} / atm	E_{app} / kJ·mol^{-1}	ln (A_{app} / s^{-1})
Fresh B-MFI	0.15	103	5.8
Fresh B-MFI	0.25	125	9.1
Activated B-MFI	0.15	142	11.7
Activated B-MFI	0.25	163	15.3

Table S6. Crystallographic data of steamed and activated B-MFI samples. The mass loadings and B atoms per unit cell are calculated from the results of ICP-AES. The lattice constants and unit cell volumes are calculated from the peak positions in XRD patterns based on the orthorhombic unit cell.

Sample	B / wt%	B/unit cell	*a* / Å	*b* / Å	*c* / Å	V_{cell} / Å^3
Fresh B-MFI	0.18	1.0	20.07	19.88	13.39	5342
B-MFI after 1 h steaming	n.d.	n.d.	20.07	19.87	13.38	5336
B-MFI after 2 h steaming	n.d.	n.d.	20.06	19.86	13.38	5330
B-MFI after 4 h steaming	n.d.	n.d.	20.07	19.87	13.38	5336
B-MFI after 12 h steaming	n.d.	n.d.	20.07	19.87	13.38	5336
Activated B-MFI	0.17	1.0	20.06	19.87	13.38	5333

Table S7. ^{11}B NMR peak fitting results of fresh, steamed and activated B-MFI zeolites.

	B[4]-Fr / %	B[3]-Fr / %	B[3]-Nf / %
Fresh B-MFI	92	8	0
B-MFI after 1 h steaming	75	25	0
B-MFI after 2 h steaming	60	35	5
B-MFI after 4 h steaming	54	35	11
B-MFI after 12 h steaming	49	36	15
Activated B-MFI	45	41	14

Table S8. Morphological properties of fresh B-BEA.

Surface area / $m^2 \cdot g^{-1}$	Pore volume / $cm^3 \cdot g^{-1}$	Micropore area / $m^2 \cdot g^{-1}$	Micropore volume / $m^2 \cdot g^{-1}$
485	0.26	437	0.22

Table S9. ^{11}B NMR peak fitting results of fresh and activated B-BEA zeolites.

	B[4]-Fr / %	B[3]-Fr / %	B[3]-Nf / %
Fresh B-BEA	64	29	7
Activated B-BEA	49	41	10

Table S10. E_{app} and A_{app} of fresh and activated B-BEA samples.

Sample	p_{C3H8} / atm	E_{app} / kJ·mol^{-1}	ln (A_{app} / s^{-1})
Fresh B-BEA	0.15	106	5.7
Fresh B-BEA	0.25	124	8.9
Activated B-BEA	0.15	119	7.8
Activated B-BEA	0.25	171	16.7

Supplementary References

[S1] J. T. Grant, C. A. Carrero, F. Goeltl, J. Venegas, P. Mueller, S. P. Burt, S. E. Specht, W. P. McDermott, A. Chieregato, I. Hermans, *Science* **2016**, *354*, 1570-1573.

[S2] L. Shi, D. Wang, W. Song, D. Shao, W.-P. Zhang, A.-H. Lu, *ChemCatChem* **2017**, *9*, 1788-1793.

[S3] J. Tian, J. Lin, M. Xu, S. Wan, J. Lin, Y. Wang, *Chemical Engineering Science* **2018**, *186*, 142-151.

[S4] J. M. Venegas, Z. Zhang, T. O. Agbi, W. P. McDermott, A. Alexandrova, I. Hermans, *Angew Chem Int Ed Engl* **2020**, *59*, 16527-16535.

[S5] X. Zhang, R. You, Z. Wei, X. Jiang, J. Yang, Y. Pan, P. Wu, Q. Jia, Z. Bao, L. Bai, M. Jin, B. Sumpter, V. Fung, W. Huang, Z. Wu, *Angew Chem Int Ed Engl* **2020**, *59*, 8042-8046.

[S6] Q. Liu, C. Chen, Q. Liu, Y. Wu, F. Xing, C. Cheng, C. Huang, *Applied Surface Science* **2021**, *537*.

[S7] H. Zhou, X. Yi, Y. Hui, L. Wang, W. Chen, Y. Qin, M. Wang, J. Ma, X. Chu, Y. Wang, X. Hong, Z. Chen, X. Meng, H. Wang, Q. Zhu, L. Song, A. Zheng, F. S. Xiao, *Science* **2021**, *372*, 76-80.

[S8] W.-D. Lu, D. Wang, Z. Zhao, W. Song, W.-C. Li, A.-H. Lu, *ACS Catalysis* **2019**, *9*, 8263-8270.

[S9] H. Tian, Y. Liu, B. Xu, *Catalysis Today* **2023**, 114048.

[S10] J. Tian, J. Li, S. Qian, Z. Zhang, S. Wan, S. Wang, J. Lin, Y. Wang, *Applied Catalysis A: General* **2021**, *623*, 118271.

[S11] B. Yan, W.-C. Li, A.-H. Lu, *Journal of Catalysis* **2019**, *369*, 296-301.

[S12] W.-D. Lu, X.-Q. Gao, Q.-G. Wang, W.-C. Li, Z.-C. Zhao, D.-Q. Wang, A.-H. Lu, *Chinese Journal of Catalysis* **2020**, *41*, 1837-1845.

[S13] B. Qiu, F. Jiang, W.-D. Lu, B. Yan, W.-C. Li, Z.-C. Zhao, A.-H. Lu, *Journal of Catalysis* **2020**,

385, 176-182.
